# Modelling in economic evaluation of mental health prevention: current status and quality of studies

**DOI:** 10.1186/s12913-022-08206-9

**Published:** 2022-07-13

**Authors:** Nguyen Thu Ha, Nguyen Thanh Huong, Vu Nguyen Anh, Nguyen Quynh Anh

**Affiliations:** 1 Department of Health Policy and Economics, Hanoi University of Public Health, Hanoi, Vietnam; 2 Department of Health Education and Promotion, Hanoi University of Public Health, Hanoi, Vietnam; 3 Hanoi University of Public Health, Hanoi, Vietnam

**Keywords:** Decision-analytic models, Economic evaluation, Value-for-money, Cost-effectiveness, Prevention, Mental health, Mental disorders, Universal prevention

## Abstract

**Background:**

The present study aimed to identify and critically appraise the quality of model-based economic evaluation studies in mental health prevention.

**Methods:**

A systematic search was performed on MEDLINE, EMBASE, EconLit, PsycINFO, and Web of Science. Two reviewers independently screened for eligible records using predefined criteria and extracted data using a pre-piloted data extraction form. The 61-item Philips Checklist was used to critically appraise the studies. *Systematic review registration number***:** CRD42020184519.

**Results:**

Forty-nine studies were eligible to be included. Thirty studies (61.2%) were published in 2015–2021. Forty-seven studies were conducted for higher-income countries. There were mainly cost-utility analyses (*n* = 31) with the dominant primary outcome of quality-adjusted life year. The most common model was Markov (*n* = 26). Most of the studies were conducted from a societal or health care perspective (*n* = 37). Only ten models used a 50-year time horizon (*n* = 2) or lifetime horizon (*n* = 8). A wide range of mental health prevention strategies was evaluated with the dominance of selective/indicate strategy and focusing on common mental health problems (e.g., depression, suicide). The percentage of the Philip checkilst’s criteria fulfilled by included studies was 69.3% on average and ranged from 43.3 to 90%. Among three domains of the Philip checklist, criteria on the model structure were fulfilled the most (72.1% on average, ranging from 50.0% to 91.7%), followed by the data domain (69.5% on average, ranging from 28.9% to 94.0%) and the consistency domain (54.6% on average, ranging from 20.0% to 100%). The practice of identification of ‘relevant’ evidence to inform model structure and inputs was inadequately performed. The model validation practice was rarely reported.

**Conclusions:**

There is an increasing number of model-based economic evaluations of mental health prevention available to decision-makers, but evidence has been limited to the higher-income countries and the short-term horizon. Despite a high level of heterogeneity in study scope and model structure among included studies, almost all mental health prevention interventions were either cost-saving or cost-effective. Future models should make efforts to conduct in the low-resource context setting, expand the time horizon, improve the evidence identification to inform model structure and inputs, and promote the practice of model validation.

**Supplementary Information:**

The online version contains supplementary material available at 10.1186/s12913-022-08206-9.

## Introduction

Mental disorders have posed a significant burden on health and wellbeing for individuals, families and communities worldwide. It is estimated that the burden of mental health disorders accounted for 14.4% of years lived with disability (YLDs) and 4.9% of disability-adjusted life years (DALYs) in 2017 [1]. An increasing body of literature discusses the benefits of interventions to promote better mental health and well-being and prevent mental illness from early childhood and adolescence until older age [2–4]. Even in high-income countries, mental health prevention interventions have not received adequate investment despite their profound benefit [2]. In the context of scarce resources, evidence on the burden of mental health and the effectiveness of mental health prevention is not adequate to advocate for the investment in mental health prevention [3, 5]. Economic evaluation tools play a more critical role in informing investment decision making both for mental health in particular and for health care in general [3].

Some systematic reviews of economic evaluations related to mental health prevention [5–9] were published, but none of them was dedicated to a model-based design. In general, the trial-based approach was the dominant study design in the previous systematic reviews. Trial-based economic evaluation might have several limitations, such as having inadequate patient follow-up and not capturing the final health outcome. Meanwhile, preventive interventions are expected to have a beneficial impact on mental health outcomes for some considerable period after the end of the trial [10]. Thus, model-based design is fundamental in an economic evaluation of mental health prevention due to its advantages, including the ability to: (1) consider all relevant alternatives required by policy makers; (2) make the results applicable to the decision-making context; (3) reflect all relevant evidence that not often collected in trials; (4) ability to reflect the final outcomes rather than intermediate outcome; (5) ability to extrapolate over medium- and long-term horizon of the evaluation. Model-based economic evaluation is also less costly than its counterpart employing trial-based design. However, poor practice in economic evaluation modelling of mental health prevention might deliver unreliable results and create barriers in disseminating the results to policymakers.

Thus, the primary objective of this study is to identify and critically appraise all model-based economic evaluations of mental health prevention interventions. This study will reveal the current situation of applying modelling techniques in the economic evaluations of mental health preventions. It will support practice and policy with evidence on the medium and long-term cost-effectiveness of mental health prevention along with the quality of evidence. This study also helps to make recommendations about future models in the field.

## Methods

We followed the Cochrane Collaboration guideline of conducting a systematic review for economic evidence [11] and consulted with other recommendations [12–14] (See Table S1-Online Supplementary file for the Prefered Reporting Items for Systematic reviews and Meta-Analysis (PRISMA) checklist). We registered the review protocol on the International Prospective Register of Systematic Reviews (CRD42020184519).

### Inclusion and exclusion criteria

The studies were included if meeting the following criteria presented in Table 1. There are many definitions relating to mental health prevention activities. This review considered the definition used by WHO [15]. Prevention of mental disorders could be categorised as universal prevention (i.e., targeting the general public or a whole population group); selective prevention (i.e., targeting subgroups of the population whose risk of developing a mental disorder is significantly higher than that of the rest of the population) and indicated prevention (i.e., targeting persons at high-risk for mental disorders). We included interventions that addressed mental disorders, such as depression, anxiety disorder, bipolar disorder, schizophrenia and other psychoses, based on ICD-10 classification [16]; or well-known mental health risks behaviours, including bullying victimisation, intimate partner violence, childhood sexual abuse and suicide. Due to the differences in the nature of prevention for mental health disorders resulting from substance abuse, dementia and other neurocognitive disorders, we excluded interventions addressing the above mental disorders.

**Table 1:** Inclusion and exclusion criteria

	Inclusion criteria	Exclusion criteria
Population	No restriction on participant characteristics such as gender, age, ethnic or country	
Intervention	Included preventive interventions in the field of mental health (included interventions on well-known mental health risks behaviours)	Interventions addressing mental disorders due to substance use, dementia or neurocognitive disorders; involving the use of drug therapy
Comparision	No restriction on the types of the comparator(s). The comparator can be either no intervention or another intervention	
Outcome	There were no restrictions on study outcomes. Potential relevant outcomes are DALYs, QALYs, effectiveness outcomes such as depression score	
Design of study	Full economic evaluations, e.g., cost-effectiveness analysis (CEA), cost-utility analysis (CUA), cost–benefit analysis (CBA) and return-on-investment (ROI); Model-based economic evaluation, i.e., comparing the expected costs and consequences of decision options by synthesising information from multiple sources and applying mahtematical techniques	Trial-based economic evaluations; partial economic evaluations; systematic reviews; case studies; commentaries; editorials; letters; conference abstracts; research protocols; animal studies
Other criteria	No restrictions based on perspective, follow-up duration, sample size, setting or time of publication	Full-text is not in English

We only included full economic evaluations, which addressed the identification, measurement, valuation and comparison of both costs and consequences of at least two alternatives [17]. We only included studies employing model-based design, which compares the expected costs and consequences of decision options by synthesising information from multiple sources and applying mathematical techniques [17, 18] (i.e., including any study beyond the direct application of observed data).

### Information sources

The following electronic bibliographic databases of published studies were searched: MEDLINE (via Pubmed), EMBASE (via http://www.embase.com), EconLit, PsycINFO and Web of Science. We also identified potential additional studies by citation tracking in Google Scholar and systematic scanning of the reference lists of eligible studies and relevant review articles. We re-performed the search on 8th November 2021.

### Search strategy and data management

The search query referred to terms covering the core concept of the research question, including mental health AND prevention/promotion intervention AND economic evaluation. We consulted the search strategy developed in a recent systematic review [8] to finalize our search strategy. Full details are available in Online Supplementary File (Table S2). The literature search results were managed using Endnote X9.

### Selection process

Two reviewers (NTH and NQA) independently screened titles and abstracts against the selection criteria. Then, all potential full-text papers were reviewed. Any disagreement or conflicting views between the two reviewers were resolved by discussion with a third reviewer (NTHg). To aid the study selection and analysis of non-English language articles, translation, either in part or in whole, will be undertaken by an appropriately qualified person.

### Data extraction

All recommended items [14], including general background, method and results of the studies, were recorded using Excel in a pre-piloted data extraction form. Two reviewers (NTH and NQA) extracted the data. Any discrepancies between the reviewers over the data extraction process were identified and resolved by discussion or the final judgement of a third reviewer (NTHg). The CCEMG-EPPI-Centre Cost Converter [19], a web-based tool, was used to adjust cost estimation into 2021 USD dollars (using International Monetary Fund World Economic Outlook Database for Purchasing Power Parities values).

### Quality assessment of included studies

Since this review focuses on modelling studies, the Philips Checklist [20] was used as recommended [21, 22]. The 61-item Philips Checklist was completed by two reviewers (NTH and NQA). Any disagreements were discussed until a consensus was reached. Responses for the checklist items included yes (Y), no (N), not applicable (N/A, for items that were not relevant to the study), and partial (P, for items that had multiple elements and were not fully satisfied by the study). To summarize the quality assessment results, we calculated the percentage of criteria fulfilled as applied by other researchers. A “Y”, “N”, “P”, and “N/A” responses were counted as one, zero or half of a point and discounted from the calculation, respectively.

### Data synthesis

Following guidance on narrative synthesis in systematic reviews [23], we employed textual descriptions, tabulation, groupings and vote-counting to synthesise the findings. Due to the heterogeneity, we used the dominance ranking matrix [24] to summarize cost-effectiveness results.

## Results

### Study selection

**Fig. 1:**
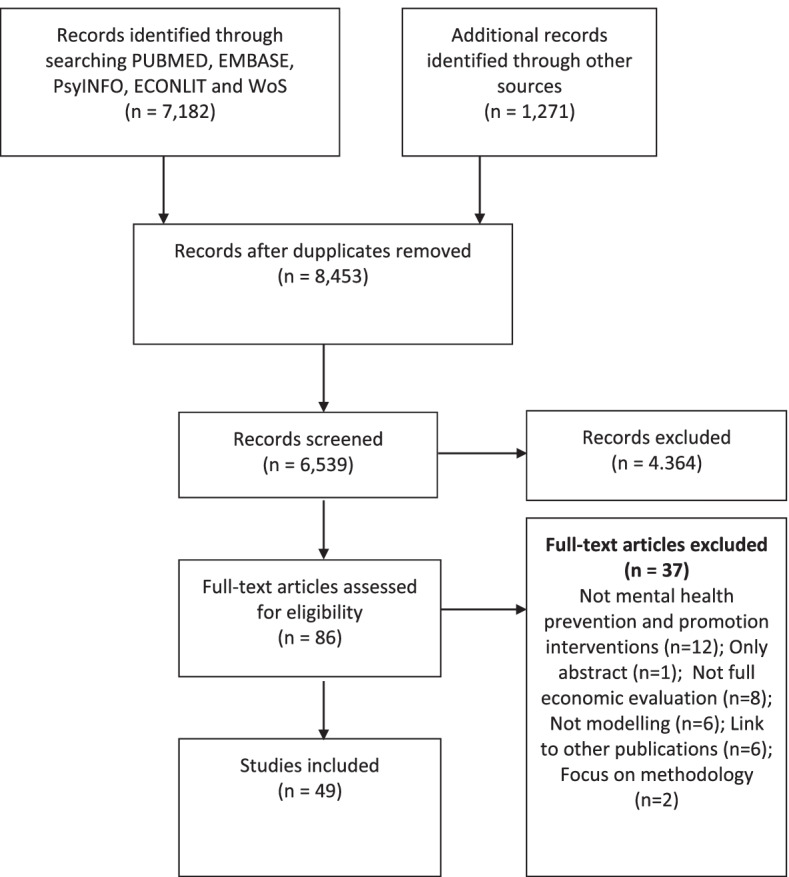
PRISMA flow diagram of study identification and selection process

The systematic search returned 8,453 records. After removing duplicates and initial screening, 86 full texts were accessed. Thirty-seven full texts were excluded (See detailed reasons for exclusion in Table S3-Online Supplementary File). Forty-nine studies were included in the review (See Fig. 1 for the selection process).

### Study characteristics

Table 2 summarises the characteristics of included studies. A wide range of mental health disorders and risk factors were evaluated in 49 included studies. Depression was the most common topic (*n* = 14), follow by suicide (*n* = 12), eating disorder (*n* = 4), anxiety (*n* = 4), bullying (*n* = 4), violence (*n* = 4), behavior disorder (*n* = 3), abuse (*n* = 3), and one exceptional study [25] on prevention of psychotic disorders for ultra-high risk population. The most common prevention approach across the studies was the indicated strategy, i.e., that targets high-risk populations (*n* = 31), followed by universal preventions (*n* = 15) and selective preventions (*n* = 10). Comparators were mainly “no intervention” or “usual care”.

**Table 2:** Summary of included studies

MHDs and risk factors	Year	Country	Type of EE	Primary Outcome measured	Perspective	Type of Intervention	Primary beneficence group	Type of Model	Time Horizon	Study performance (min; max)
Depression (*n* = 14)	2001 (1) 2010–14 (8) 2015–21 (5)	Aus (3), US (3), UK (2), NL (2), Nor (1), Swe (1), Can (1), other (1)	CUA (12), CEA (2), CBA (1), ROI (1)	QALY (8), DALY (5), monetary (2), cases (1)	Societal (5), health (7), education (1), payer (2), other sector (1), not stated (1)	Universal (3), Indicated (12), Selective (1)	Adult (10), Children& adolescent (4)	Markov (9), Decision tree (2), Markov + Decision tree (1), Unclear (2)	$\ge$ 10 years (4); 5–9 years (5); < 5 years (5)	(48%; 83%)
Eating Disorder (*n* = 4)	2011–14 (2) 2017 (2)	US (3), Aus (1)	CUA (3), CEA (2)	QALY (2), DALY (1), LY (1), case (1)	Societal (1), health (1), payer (2)	Universal (1), Indicated (2), Selective (2)	Children& adolescent (4)	Markov (2), Unclear (2)	$\ge$ 10 years (3); < 5 years (1)	(44%; 78%)
Anxiety (*n* = 4)	2013 (1) 2015–18 (3)	NL (2), US (1), Aus (1)	CUA (3), CEA (1)	QALY (2), DALY (1), cases (1)	Societal (3), health (1)	Indicated (3), Selective (1)	Adult (2), children& adolescent (2)	Markov (2), Decision tree (1), Unclear (1)	$\ge$ 10 years (1); 5–9 years (1); < 5 years (2)	(56%; 82%)
Behavior Disorder (*n* = 3)	2007 (1) 2019–20 (2)	Swe (2), Aus (1)	CUA (1), CEA (1), CBA (1)	DALY (1), monetary (1), case (1)	Societal (1); health (2), eduation (1), other sectors (1)	Universal (1), Indicated (2)	Children& adolescent (3)	Markov (2), Unclear (1)	$\ge$ 10 years (3)	(65%; 72%)
Phsychotic disorder (*n* = 1)	2020 (1)	NL (1)	CUA (1)	QALY (1)	Health (1)	Selective (1)	Adult (1)	Markov (1)	$\ge$ 10 years (1)	(90%)
Suicide (*n* = 12)	2013 (3) 2015–21 (8)	US (4), Aus (2), Sri Lanka (1), Bel (1), Can (2), Spain (1), other (1)	CUA (5), CEA (4), CBA (1), ROI (2)	QALY (3), DALY/HLYG (2), LY (3), monetary (3), case (1)	Societal (7), health (3), other sector (1), payer (1), not stated (1)	Universal (5), Indicated (8), Selective (2)	Adult (10), children& adolescent (3)	Markov (6), Decision tree (1), Unclear (5)	$\ge$ 10 years (5); 5–9 years (1); < 5 years (6)	(43%; 86%)
Bullying (*n* = 4)	2009 (1) 2015–19 (3)	Swe (2), NL (1), UK (1)	CUA (2), CEA (2), CBA (1)	QALY (2), LY (2), monetary (1)	Societal (1), payer (2), not stated (1)	Universal (4), Indicated (3), Selective (1)	Children& adolescent (4)	Markov (1), Decision tree (1), Unclear (2)	$\ge$ 10 years (2); 5–9 years (1); < 5 years (1)	(68%; 77%)
Violence (*n* = 4)	2010–13 (3) 2018 (1)	UK (4)	CUA (4)	QALY (4)	Societal (3), payer (1)	Indicated (4)	Adult (4)	Markov (3), Decision tree (1)	$\ge$ 10 years (3); < 5 years (1)	(71%; 84%)
Abuse (*n* = 3)	2018–20 (3)	US (3)	CBA (3)	Monetary (3)	Societal (3)	Universal (1) Indicated (1) Selective (1)	Children& adolescent (3)	Unclear (3)	$\ge$ 10 years (3)	(61%; 77%)
Total (*n* = 50)	2001–9 (3) 2010–14 (17) 2015–21 (29)	UMHICs (48) LLMICs (2)	CUA (31), CEA (13), CBA (7), ROI (3)	QALY (21), DALY/HLYGs (10), monetary (11), LY (6), cases (5)	Societal (22), health (15), education (5), payer (8), other sector (2), not stated (3)	Universal (15), Indicated (31), Selective (9)	Adult (27), Children& adolescent (23)	Markov (26), Decision tree (6), Markov + Decision tree (1), Unclear (16)	$\ge$ 10 years (25); 5–9 years (8); < 5 years (16)	(43.3%; 90.0%)

NB: The total number of included studies in each category might exceed 50 since one might have more than one characteristic

*EE* Economic evaluation, *NL* The Netherlands, *UK* The United Kingdom, *US* The United States, *AUS* Australia, *Nor* Norway, *Swe* Sweden, *LLMICs* Low-income and lower-middle-income countries, *UMHICs* Upper-middle-income and high-income countries

The included studies were published from 2001 to 2021. Only three [26–28] studies were published before 2010, with the earliest one on depression published in 2001 [27]. From 2010 until 2014, 17 studies were published. Almost double this number of studies (*n* = 29) were published in 2015–2021. The majority of models (*n* = 47) were conducted for higher-income countries. Meanwhile, only one study was conducted in Sri Lanka [29], a lower-middle-income country, and another study [30] was performed in multiple countries, including both higher-income and lower-income countries. Regarding the type of economic evaluation, there were 26 CUAs, nine CEAs, six CBAs and three ROIs and the remaining studies were a combination of CEA and CUA (*n* = 4) or CUA and CBA (*n* = 1). For the CUAs, Quality-Adjusted Life Year (QALY) was most commonly used (*n* = 21). In ten studies, Disability-Adjusted Life Year (DALY) and its variant (Healthy-Life Year Gained, HLYG) were used. The clinical outcomes measuring in the CEAs included life-year (LY) gained [29, 31, 32], life year with a mental health problem (i.e., eating disorder) avoided [33], victim-free year (for bullying) [34, 35], cases (i.e., cases with behaviour disorder [26], eating disorder [36], depression [37], and suicide [38]) or cases with meaningful change on symptom scale [39].

A societal perspective was taken in 22 studies, followed by 15 studies that took the health sector perspective. Three studies did not state the perspective used [28, 31, 40]. Markov models were the most common modelling approach, used in 26 studies (52.0%). Other six studies employed decision tree [35, 38, 39, 41–43], and one study employed a combination of Markov and decision tree [44]. The remaining 16 studies did not explicitly describe their model type. They simply applied mathematic formulations without figures presenting their model structure. Their so-called modelling approach could not be classified under any paradigm (i.e. cohort-bassed like Markov, decision tree, system dynamics model or individual-based like discrete event simulation, agent-based model).

### Quality assessment

The detailed quality assessment results using Philips Checklist for each study are presented in Table 3. As proposed in the method part, we applied a scoring system to estimate the percentage of the number of Philips Checklist’s items fulfilled (i.e., applied one, zero, half of a point and discounted from the calculation for the “Y”, “N”, “P”, and “N/A” responses, respectively). As a result, the scores from this calculation were 69.3% on average and ranged from 43.3% to 90.0% for overall study performance. Among three domains of the Philip checklist, criteria on model structure were fulfilled the most (72,1% on average, ranging from 50,0% to 91,7%), followed by the data domain (69,5% on average, ranging from 28,9% to 94,0%) and the consistency domain (54,6% on average, ranging from 20,0% to 100%). The following parts present the results of quality appraisal in terms of three domains of the Phillips Checklist, i.e., model structure, data and consistency.

**Table 3:** Quality assessment results using the Phillips Checklist

**Study**	**Phillips Items 1–31**																														
	**1**	**2**	**3**	**4**	**5**	**6**	**7**	**8**	**9**	**10**	**11**	**12**	**13**	**14**	**15**	**16**	**17**	**18**	**19**	**20**	**21**	**22**	**23**	**24**	**25**	**26**	**27**	**28**	**29**	**30**	**31**
Lee (2017) [45]	y	y	n	y	p	y	y	y	y	n	y	y	p	y	y	y	y	y	y	y	n	Y	y	p	y	y	y	y	y	y	y
Mihalopoulos (2012) [7]	y	y	y	y	y	y	y	y	p	n	y	y	p	y	y	y	y	y	p	p	n	N	y	p	y	y	y	y	p	NA	y
Mihalopoulos (2011) [46]	y	y	y	y	y	y	y	n	y	n	n	y	p	y	y	y	y	y	y	y	n	Y	y	p	y	y	y	y	y	NA	y
Lokkerbol (2014) [47]	y	y	y	y	y	y	y	y	y	n	y	y	y	y	y	y	y	y	n	p	n	N	y	n	y	NA	y	y	n	y	y
van den Berg (2011) [48]	y	y	n	y	y	y	y	n	n	n	n	y	p	p	y	y	n	y	n	p	n	y	y	p	y	NA	y	n	n	NA	y
Hunter (2014) [44]	y	y	n	y	p	p	y	y	y	n	y	n	p	n	n	y	n	y	n	p	n	n	y	p	y	y	y	y	y	NA	y
Paulden (2010) [41]	y	y	y	y	p	y	y	y	y	n	p	y	y	p	y	y	n	n	n	p	n	n	y	NA	y	y	y	y	n	n	y
Goetzel (2014) [49]	y	y	y	n	NA	y	y	y	y	n	y	y	y	y	y	y	n	y	n	y	n	y	NA	NA	y	y	y	y	y	NA	y
Jiao (2017) [50]	y	y	y	y	y	y	y	y	y	n	y	y	y	y	y	y	n	y	y	n	y	NA	y	n	y	y	y	n	y	NA	y
Lintvedt (2013) [40]	y	y	n	n	y	y	y	n	p	n	n	n	n	y	y	n	NA	y	y	n	n	y	NA	NA	y	y	y	n	y	NA	y
Valenstein (2001) [27]	y	y	y	y	y	y	y	n	y	n	n	y	y	y	y	y	n	y	y	y	y	NA	y	y	y	y	y	y	y	NA	y
Ssegonja (2020) [37]	y	y	y	y	y	y	y	n	y	n	n	y	y	y	y	y	n	y	y	y	n	y	y	y	y	y	y	y	y	NA	y
Feldman (2020) [51]	y	y	n	y	y	y	y	n	y	n	n	y	y	y	y	y	n	y	y	y	n	n	y	y	y	y	y	y	y	NA	y
Premji (2021) [42]	y	y	y	y	y	y	y	p	y	n	n	y	y	y	y	n	n	y	n	y	n	y	NA	NA	y	NA	y	y	y	n	y
Le (2017) [52]	y	y	y	y	y	y	y	y	p	n	y	y	y	p	y	y	y	y	y	y	n	n	y	n	y	y	y	y	y	n	y
Wright (2014) [33]	y	y	y	y	y	y	y	y	y	n	y	y	y	y	y	y	n	y	y	y	n	y	y	y	y	y	y	y	y	NA	y
Wang (2011) [53]	y	y	y	y	y	y	y	n	y	n	n	y	y	y	y	y	n	n	y	y	n	n	NA	NA	y	y	y	y	y	NA	y
Kass (2017) [36]	y	y	y	y	y	y	y	n	p	n	n	n	y	y	y	y	n	n	n	n	n	n	NA	NA	y	y	y	n	n	NA	y
Simon (2013) [39]	y	y	y	y	y	y	y	n	n	n	n	y	y	y	y	y	n	n	n	p	n	y	y	NA	y	NA	y	n	n	NA	y
Mihalopoulos (2015) [54]	y	y	y	y	y	y	y	n	y	n	n	y	y	y	y	y	y	n	y	y	n	y	n	NA	y	y	y	n	y	NA	y
Ophuis (2018) [55]	y	y	y	y	y	y	y	y	y	n	n	y	y	y	y	y	n	y	p	p	n	n	y	y	y	y	y	p	y	n	y
Kumar (2018) [56]	y	y	n	y	y	y	y	y	y	n	y	y	y	y	y	y	n	y	y	y	y	NA	y	y	y	y	y	y	y	n	y
Mihalopoulos (2007) [26]	y	y	y	y	y	y	y	n	p	n	n	n	y	y	y	y	n	n	y	p	n	y	NA	NA	y	y	y	n	y	NA	y
Nystrand (2020) [57]	y	y	y	y	y	y	p	n	p	n	n	y	p	p	y	y	n	y	y	y	n	y	y	p	y	y	y	n	p	NA	y
Nystrand (2019) [58]	y	y	y	y	p	y	p	n	p	n	n	y	p	p	y	y	n	y	y	y	n	y	p	p	y	y	y	n	p	NA	y
Wijnen (2020) [25]	y	y	y	y	y	y	y	y	y	n	y	y	y	y	y	y	y	y	y	y	n	y	y	y	y	y	y	y	y	y	y
Lebenbaum (2020) [59]	y	y	y	y	p	y	p	y	p	n	y	p	y	p	y	y	n	y	y	y	y	NA	p	y	y	y	y	y	y	NA	y
Pil (2013) [60]	y	y	n	y	p	p	y	n	p	n	n	y	p	p	y	y	n	y	y	p	n	n	y	p	y	y	y	n	n	NA	p
Denchev (2018) [31]	y	y	n	n	p	y	y	y	y	n	n	y	p	y	y	y	y	y	n	n	n	n	y	p	y	y	y	p	p	n	y
Comans (2013) [61]	y	y	n	y	p	y	y	y	y	n	y	y	p	y	n	y	n	y	n	n	n	n	y	p	y	n	n	n	n	NA	y
Godoy (2018) [62]	y	y	y	y	y	y	y	n	y	n	n	y	y	y	y	y	n	y	n	y	n	y	NA	NA	y	y	y	n	y	NA	y
Vasiliadis (2015) [32]	y	y	y	y	y	y	y	n	y	n	n	y	n	y	y	n	n	y	n	n	n	n	NA	NA	y	y	y	n	y	NA	y
Atkins (2013) [63]	y	y	n	y	y	y	y	n	y	n	n	y	y	n	y	y	n	n	y	n	n	NA	NA	NA	y	y	y	n	n	NA	n
Damerow (2020) [29]	y	y	y	y	y	y	p	y	n	n	y	y	y	n	y	y	n	n	y	n	n	y	NA	NA	y	y	n	n	n	n	n
Kinchin (2020) [64]	y	y	n	y	y	y	y	n	y	n	n	y	y	y	y	y	n	y	y	y	n	y	y	y	y	y	y	y	y	n	y
Richardson (2017) [65]	y	y	y	y	y	y	y	n	p	n	n	y	y	y	y	y	n	y	n	y	n	y	NA	NA	y	y	y	y	n	NA	y
Lee (2020) [30]	y	y	y	y	y	y	y	y	y	n	y	y	y	y	y	y	n	y	y	y	y	NA	p	y	y	y	y	y	y	y	y
Martínez-Alés (2021) [38]	y	y	y	y	y	y	y	n	y	n	n	y	y	y	y	y	n	y	n	y	n	n	NA	NA	y	n	n	n	y	NA	y
Persson (2018) [34]	y	y	n	y	n	p	p	n	y	n	n	y	y	y	y	y	n	y	n	y	n	y	p	p	y	y	y	y	y	NA	y
Hummel (2009) [28]	y	y	n	n	y	y	y	y	y	n	y	y	y	y	y	y	n	y	y	y	y	NA	NA	NA	y	y	y	y	y	NA	y
Beckman (2015) [35]	y	y	n	y	y	y	y	p	y	n	y	y	y	y	y	y	y	y	n	y	n	y	y	NA	y	y	y	y	y	NA	y
Huitsing (2019) [66]	y	y	y	y	y	y	y	n	y	n	n	y	y	y	y	y	y	y	y	y	y	NA	NA	NA	y	y	y	n	y	n	y
Devine (2012) [67]	y	y	y	y	y	y	y	y	y	n	y	y	y	y	y	y	n	y	y	y	n	y	y	y	y	y	y	y	y	NA	y
Mallender (2013) [43]	y	y	y	y	y	y	y	y	y	n	y	y	y	y	y	y	y	y	n	y	n	y	y	NA	y	y	y	y	y	NA	y
Norman (2010) [68]	y	y	n	y	y	y	y	n	y	n	n	y	y	y	y	n	n	y	y	y	n	y	y	y	y	NA	y	p	y	NA	y
Barbosa (2018) [69]	y	y	n	y	y	y	y	y	y	n	y	y	y	n	y	y	n	y	y	n	n	n	y	y	y	y	y	y	y	NA	y
Dopp (2018) [70]	y	y	y	y	y	y	y	y	y	n	y	y	p	p	y	y	n	y	y	y	y	NA	NA	NA	y	y	y	y	y	NA	y
Peterson (2018) [71]	y	y	y	y	y	y	y	n	n	n	n	y	y	p	y	y	y	n	y	p	y	NA	NA	NA	y	y	y	n	y	NA	y
Kuklinski (2020) [72]	y	y	n	y	y	y	y	y	y	n	y	y	p	p	y	y	n	y	y	y	y	NA	NA	NA	y	y	y	p	y	NA	y
**Study**	**Phillips Items 32–61**																														**Overall performance**
	**32**	**33**	**34**	**35**	**36**	**37**	**38**	**39**	**40**	**41**	**42**	**43**	**44**	**45**	**46**	**47**	**48**	**49**	**50**	**51**	**52**	**53**	**54**	**55**	**56**	**57**	**58**	**59**	**60**	**61**	
Lee (2017) [45]	y	y	n	n	y	y	y	y	y	y	y	y	y	y	y	p	y	y	NA	y	y	y	y	y	y	n	y	n	n	y	83%
Mihalopoulos (2012) [7]	y	y	n	n	y	y	y	y	y	y	y	n	y	p	p	p	y	y	NA	y	y	y	y	y	y	n	y	n	n	y	77%
Mihalopoulos (2011) [46]	y	y	n	n	y	y	y	y	y	y	y	n	y	p	p	p	y	y	NA	y	y	y	y	y	y	n	y	y	n	y	81%
Lokkerbol (2014) [66]	y	y	n	n	y	y	y	y	y	y	n	n	p	p	p	y	y	n	n	y	y	n	y	y	y	n	y	y	n	y	72%
van den Berg (2011) [48]	y	y	n	n	n	y	p	y	n	y	y	y	y	p	y	p	y	n	n	n	n	n	y	y	y	n	y	y	n	n	57%
Hunter (2014) [44]	p	y	y	NA	y	NA	NA	NA	NA	y	y	y	y	p	y	p	y	y	NA	y	y	y	y	y	y	n	y	y	n	n	70%
Paulden (2010) [41]	p	y	NA	NA	y	NA	NA	NA	NA	y	y	y	y	y	y	p	y	y	NA	y	y	y	y	y	y	n	y	y	n	n	74%
Goetzel (2014) [49]	y	NA	NA	NA	NA	y	n	NA	NA	NA	NA	NA	p	y	y	NA	NA	n	n	n	n	n	n	n	n	n	y	y	n	n	62%
Jiao (2017) [50]	y	y	n	n	n	n	y	n	n	y	y	y	y	y	y	y	y	n	n	n	n	n	y	y	y	n	y	y	n	n	68%
Lintvedt (2013) [40]	y	NA	NA	NA	y	y	n	n	n	n	n	n	p	y	y	NA	NA	n	n	y	n	n	y	n	n	n	y	y	n	n	48%
Valenstein (2001) [27]	y	y	n	n	p	y	y	y	y	y	y	y	y	y	y	p	y	n	n	y	y	n	y	y	y	n	y	y	n	y	80%
Ssegonja (2020) [37]	y	y	n	n	y	y	y	y	y	y	y	y	y	y	y	y	y	n	y	y	y	n	y	y	y	n	y	y	n	y	82%
Feldman (2020) [51]	n	n	n	n	y	y	y	y	y	y	y	NA	n	y	n	n	y	n	y	y	y	n	y	y	y	n	y	y	n	n	68%
Premji (2021) [42]	y	NA	NA	NA	n	NA	NA	NA	NA	y	y	y	y	y	y	y	y	n	n	y	y	n	y	y	y	n	y	y	n	y	74%
Le (2017) [52]	p	y	n	n	y	y	y	y	y	y	y	n	y	p	y	p	y	n	n	y	y	n	y	y	y	n	y	y	n	y	75%
Wright (2014) [33]	y	y	n	n	NA	y	y	y	y	y	y	y	y	y	y	y	y	n	n	n	n	n	y	y	y	n	y	y	n	n	78%
Wang (2011) [53]	y	NA	NA	NA	y	y	y	y	y	y	y	y	y	y	y	y	y	n	n	n	y	n	y	y	y	n	y	y	n	y	76%
Kass (2017) [36]	y	NA	NA	NA	y	y	n	n	n	NA	NA	NA	NA	y	y	n	n	n	n	n	n	n	n	n	n	n	y	y	n	n	44%
Simon (2013) [39]	y	y	NA	NA	NA	NA	NA	NA	NA	n	NA	NA	p	y	y	n	NA	n	n	y	y	n	y	n	p	n	p	y	n	n	56%
Mihalopoulos (2015) [54]	n	n	NA	NA	n	n	y	p	y	y	y	n	y	y	y	n	n	n	n	y	y	n	y	y	p	n	y	y	n	n	63%
Ophuis (2018) [55]	n	y	n	n	n	n	y	n	n	y	y	n	p	p	y	n	y	n	n	y	y	n	y	y	p	n	p	y	n	n	60%
Kumar (2018) [56]	y	y	n	n	y	y	y	y	y	y	y	y	y	y	y	NA	NA	n	n	n	y	n	y	n	y	y	y	y	y	y	81%
Mihalopoulos (2007) [26]	y	NA	NA	NA	n	n	y	n	n	NA	NA	NA	y	y	y	NA	NA	y	NA	y	y	y	y	n	y	n	y	y	n	n	65%
Nystrand (2020) [57]	y	y	n	n	NA	y	y	y	y	p	NA	NA	y	y	y	y	y	n	n	y	y	n	y	y	p	n	y	y	n	y	70%
Nystrand (2019) [58]	y	y	n	n	NA	y	y	y	y	y	y	p	y	p	y	p	y	y	NA	y	y	y	y	y	p	n	y	y	n	y	72%
Wijnen (2020) [25]	y	y	y	NA	n	y	y	y	y	y	y	y	y	y	y	y	y	n	n	y	y	n	y	y	y	y	y	y	y	y	90%
Lebenbaum (2020) [59]	y	y	y	NA	y	y	y	y	y	y	y	y	y	p	y	p	y	n	n	y	y	n	y	y	y	n	y	y	n	y	81%
Pil (2013) [60]	y	y	n	n	n	n	NA	n	NA	p	y	n	n	p	y	p	y	y	NA	y	y	y	y	y	p	n	y	y	n	y	58%
Denchev (2018) [31]	y	y	n	n	n	n	y	n	y	NA	NA	NA	n	n	y	n	y	n	n	n	y	n	y	y	p	n	y	n	n	n	52%
Comans (2013) [61]	y	n	y	NA	NA	p	p	n	p	y	y	y	n	n	n	p	y	n	n	n	y	n	y	y	n	n	y	p	n	n	50%
Godoy (2018) [62]	y	NA	NA	NA	NA	y	y	NA	NA	NA	NA	NA	y	y	y	NA	NA	n	n	y	y	n	y	n	y	n	y	y	n	y	72%
Vasiliadis (2015) [32]	y	NA	NA	NA	NA	NA	NA	NA	NA	NA	NA	NA	y	y	y	n	NA	n	n	y	n	n	y	n	y	n	y	y	n	y	59%
Atkins (2013) [63]	n	NA	NA	NA	NA	NA	NA	NA	NA	n	NA	NA	p	y	y	NA	NA	n	n	n	n	n	n	n	n	n	y	y	n	n	43%
Damerow (2020) [29]	n	NA	NA	NA	NA	NA	NA	NA	NA	n	NA	NA	n	n	n	NA	NA	n	n	y	y	n	y	n	y	n	y	y	n	n	48%
Kinchin (2020) [64]	y	y	y	NA	y	y	y	y	y	NA	NA	NA	y	y	y	NA	NA	n	n	y	y	n	y	n	y	y	y	y	y	y	80%
Richardson (2017) [65]	y	NA	NA	NA	NA	NA	NA	NA	NA	NA	NA	NA	y	y	y	NA	NA	n	n	n	n	n	y	y	y	n	y	y	n	y	68%
Lee (2020) [30]	y	y	n	n	y	y	y	y	y	y	n	n	y	y	y	y	y	y	NA	y	y	y	y	y	y	n	y	y	n	y	86%
Martínez-Alés (2021) [38]	y	NA	NA	NA	n	NA	y	NA	NA	NA	NA	NA	y	y	y	y	y	n	n	y	y	n	y	y	y	n	y	y	n	y	67%
Persson (2018) [34]	y	y	n	n	y	y	y	y	n	y	y	y	y	y	y	p	y	n	n	y	y	n	y	y	y	n	y	p	n	y	68%
Hummel (2009) [28]	y	NA	NA	NA	NA	y	y	y	y	y	y	y	y	y	y	n	y	n	n	y	n	n	y	y	y	n	y	y	n	n	77%
Beckman (2015) [35]	y	NA	NA	NA	n	y	y	NA	NA	NA	NA	NA	y	y	y	y	y	n	y	n	n	n	y	y	y	n	y	p	n	y	76%
Huitsing (2019) [66]	y	NA	NA	NA	NA	y	y	y	y	NA	NA	NA	y	y	y	NA	NA	n	n	y	y	n	y	n	y	n	y	y	n	n	76%
Devine (2012) [67]	y	y	n	n	NA	y	y	y	y	y	y	NA	y	y	y	y	y	n	n	y	y	n	y	y	y	y	y	y	y	n	84%
Mallender (2013) [43]	y	y	NA	NA	y	y	y	NA	NA	y	y	y	y	y	y	NA	NA	n	n	n	n	n	y	n	y	n	y	y	n	y	79%
Norman (2010) [68]	y	y	n	n	NA	y	y	y	y	y	y	y	y	y	y	NA	NA	n	n	n	n	n	y	n	y	y	y	y	y	y	72%
Barbosa (2018) [69]	y	y	y	NA	n	n	y	NA	NA	n	y	n	y	y	y	p	y	n	n	n	n	n	y	y	y	y	y	y	y	y	71%
Dopp (2018) [70]	y	NA	NA	NA	y	y	y	y	n	NA	NA	NA	y	y	y	p	y	n	n	n	n	n	y	y	y	n	y	y	n	y	77%
Peterson (2018) [71]	y	NA	NA	NA	n	y	n	y	n	NA	NA	NA	y	y	y	NA	NA	n	n	n	n	n	y	n	n	n	y	y	n	y	61%
Kuklinski (2020) [72]	y	NA	NA	NA	p	y	y	y	n	NA	NA	NA	p	y	y	y	y	n	n	y	y	n	y	y	p	n	y	y	n	y	76%

#### Model structure

Detailed information on some key structural aspects of the included models is presented in Table 4. Almost all studies demonstrated a clear statement of the decision problem and objectives of the model. However, the primary decision-maker was only specified in 33 studies (67.3%). Although the statement of scope and perspective of the models were commonly stated clearly, there were four remaining studies [28, 31, 40, 49] that did not explicitly state the studies’ perspectives.

**Table 4:** General characteristic of included studies

Author (Year) Country	EE Type (primary outcome) And perspective	Intervention *and comparator*	Model	Time Horizon (cycle)	Rationale for model structure	Model validation	Intervention Effectiveness	Data source for effectiveness	Assumption on long term effect	Sensitivity analysis	Software used for model
Depression (*n* = 14)											
Lee (2017) [45] Australia	CUA (DALY) Health, education sector	Group-based psychological intervention *No intervention*	Markov	10 years (1 year)	A simple incidence-prevalence-mortality model (DisMod2)	Prevention experts feedback on intervention coverage	Depression incidence (measured using structured clinical interviews/ depression symptom rating scale)	Meta-analysis	Effect remains 1 year	Univariate, PSA	Excel 13
Mihalopoulos (2011) [46] Australia	CUA (DALY) Health sector, payer	Opportunistic screening for Sub-syndromal depression + psychological intervention *Do-nothing*	Markov	5 years (1 year)	Unclear	Not mentioned	Depression incidence	RCT (1-year follow-up) and meta-analysis	Effect remains 2 years, from year 2–5 decay effect = 50%	PSA	Unclear
Paulden (2010) [41] UK	CUA (QALY) Health sector	Routine screening for postnatal depression + psychological therapy *Usual care*	Decision tree	1 year	Unclear	Not mentioned	Depression incidence	RCT (1-year follow-up) and meta-analysis	Effect remains 2 years, from year 2–5 decay effect = 50%	Unclear	Unclear
Hunter (2014) [44] UK	CUA (QALY) Health sector	Screening with a Risk Algorithm (PredictD) + low-intensity prevention program *Treatment as usual*	Markov + Decision tree	12 months *(3 months)*	Systematic review	Not mentioned	Depression incidence	Meta-analysis of similar preventions	No	PSA	Excel 2010
Lokkerbol (2014) [47] Netherlands	CUA, CBA (DALY, monetary) Health sector	Preventive telemedicine *Usual care*	Markov	5 years *(1 year)*	Population-based cohort data	Expert panel was used to select interventions only	Depression incidence	Meta-analysis	Effect remains 1 year	PSA	Unclear
Mihalopoulos (2012) [7] Australia	CUA (DALY) Health, other sectors	Screening + psychological intervention *Do-nothing*	Markov	5 years *(1 year)*	Prior EE model	Not mentioned	Depression incidence	Own meta-analysis (8 RCTs of similar interventions, 1-year follow-up))	Effect remains 2 years, from year 2–5 decay effect = 50%	Univariate, PSA	Unclear
van den Berg (2011) [48] Netherlands	CUA (DALY) *Societal*	Opportunistic screening + minimal contact psychotherapy Current practice	Markov	5 years (*4 weeks)*	Unclear	Not mentioned	Depression incidence	RCT (3-year follow-up)	Effect remains 1 year	PSA	Unclear
Ssegonja (2020) [37] Sweden	CEA, CUA (depression case, QALY), *Societal*	Group-based cognitive behaviour therapy (CBT) *No intervention*	Markov	5 years *(1 year)*	Unclear	Not mentioned	Depression incidence and depression symptom	Meta analysis (RCTs, 1-year follow-up)	Decay rate = 40%	Univariate, PSA	Excel
Valenstein (2001) [27] US	CUA (QALY) *Societal*	Depression Screening *No intervention*	Markov	Lifetime *(3 months)*	Unclear	Not mentioned	Screening sensitivity and specificity	Average of 9 instruments	No	Univariate, PSA	Treeage
Goetzel (2014) [49] US	ROI (monetary) Payer	Workplace health risk management program *No intervention*	Unclear	1 year	Truven Health Analytics ROI model	Not mentioned	Percentage point change in the 10 health risks (including high risk of high stress and depression)	Pre-post intervention study	No	None	Unclear
Lintvedt (2013) [40] Norway	CUA (QALY) Unclear perspective	e-CBT No intervention	Unclear	1 year	Unclear	Not mentioned	Rosser Classification of illness states scale to proximate utility	RCT	Effect remains 1 year	Univariate	Unclear
Jiao (2017) [50] US	CUA (QALY) Societal	Depression screening + collaborative care *No screening*	Markov	50 years *(1 year)*	Prior EE models	Not mentioned	Sensitivity and specificity; adequate treatment (CC)	Validation studies; RCT	Intervention runs over time horizon	Univariate, PSA	Tree- Age 2016
Feldman (2020) [51] High-income	CUA (QALY) *Societal*	Group-based cognitive behaviour therapy *No intervention*	Markov	5,10 years *(1 year)*	Unclear	Not mentioned	Depression	Meta-analysis	Decay rate of 40%	Univariate, PSA	Excel
Premji (2021) [42] Canada	CEA (QALY) Health sector	Screening for depression and follow-up diagnosis and treatment *No screening*	Decision tree	2 years	Not mentioned	Mentioned that validated with frontline care providers but not details	Sensitivity and specificity of screening tool	Systematic review	No	Univariate, PSA	Excel
Eating Disorder (*n* = 5)											
Le (2017) [52] Australia	CUA (DALY) Health sector	Cognitive dissonance intervention *No intervention*	Markov	10 years *(1 year)*	A simple incidence-prevalence-mortality model (DisMod2)	Not mentioned	ED symptoms measured by EDDS/EDDI	Meta-analysis (Le et al., 2017)	Effect remains 4 years with decay rate = 50%	Univariate, PSA	Excel 2010
Kass (2017) [36] US	CEA (ED case) Payer	Screening + online preventive or treatment *Wait list control*	Unclear	2 years	Not mentioned	Not mentioned	ED symptoms; ED incidence	Systematic reviews, RCT, pre-post intervention study	No	None	Unclear
Wang (2011) [53] US	CUA (QALY) Societal	School-based education and physical activity (Planet Health) *Usual curricula*	Unclear	10 years	Unclear	Not mentioned	Disordered weight control behaviors (DWCB)	RCT (Planet Health)	No	Univariate, PSA	Unclear
Wright (2014) [33] US	CEA, CUA (LY with ED, QALY), Payer	School-based eating disorder screening *No screening*	Markov	10 years *(1 year)*	Literature review	Representatives from the National Eating Disorders Association (only for interventions)	Screening sensitivity and specificity	A single study (104 primary care attendants, 129 university students)	Intervention run over time horizon	Univariate, PSA	TreeAge
Anxiety (*n* = 4)											
Ophuis (2018) [55] Netherlands	CUA (QALY) Societal	CBT-based early intervention for subthreshold panic disorder *Usual care*	Markov	5 years *(1 year)*	Intervention clinical evidences (Meulenbeek et al., 2010 and Smit et al., 2009) and available epidemiology data; expert opinion (not detailed)	Not mentioned	Clinically significant change on the Panic Disorder Severity Scale–Self Report (PDSS–SR)	Effect size of PD treatment based on meta-analysis for anxiety, of CBT based on RCT	Effect remains 5 years	PSA	Excel 2013
Mihalopoulos (2015) [54] Australia	CUA (DALY) Health sector	Screening and parenting educational program *Do-nothing*	Unclear	3 years	Unclear	Not mentioned	Proportions of children with one or more anxiety diagnoses	RCT (3-year follow-up)	No	PSA	Unclear
Simon (2013) [39] Netherlands	CEA (symptom improved child) Societal	Screening + early child/parental focused intervention *Do nothing*	Decision tree	2 years	Unclear	Not mentioned	Presence and severity of anxiety diagnoses in the children using the Anxiety Disorder Interview Schedule (ADIS)	RCT	No	Univariate	Treeage Pro 2012
Kumar (2018) [56] US	CUA (QALY) Societal	Mobile CBT *No CBT or traditional CBT*	Markov	Lifetime *(3 months)*	prior EE models	Not mentioned	Clinically response to CBT	Systematic review	Effect remains lifetime with a time based linear function of waning effect	Univariate	TreeAge Pro 2016
Behaviour Disorder (*n* = 3)											
Nystrand (2020) [57] Sweden	CBA (monetary) Societal	Group-based indicated parenting programs *Wait list control*	Markov	Until 20 years old (1 year)	Unclear	Not mentioned	Recovered cases (changes in parent reported ADHP (SNA-IV scale) and CP (ECBI scale)	RCT (original intervention, 2-year follow-up)	Effect remains 2 years	Univariate, PSA	Excel 2016
Nystrand (2019) [58] Sweden	CUA (DALY) Health, education sector	Group-based indicated parenting programs *Wait list control*	Markov	Until 18 years old *(1 year)*	Unclear	Not mentioned	Recovered cases (changes in parent reported ADHP (SNA-IV scale) and CP (ECBI scale)	RCT (original intervention, 2-year follow-up)	Effect remains after 2 years with decay rate = 50%	Univariate, PSA	Excel
Mihalopoulos (2007) [26] Australia	CEA (disruptive behaviour case) Health, other sectors	Multi-level system of parenting and family support (Triple P) *No intervention*	Unclear	1 year	unclear	Not mentioned	Parent reported of disruptive behaviour in children (ECBI scale) and parent daily report (PDR)	2 RCTs (up to 3-year follow-up) of similar interventions;	No	Univariate	Unclear
Psychosis (*n* = 1)											
Wijnen (2020) [25] Netherlands	CUA (QALY) Health sector	CBT-based intervention *Usual care*	Markov	10 years *(1 year)*	A disease classification, expert panel	Face validation (health economics experts); internal validation (extreme value testing); cross validity testing (e.g. to other staging and health economic models)	Psychosis averted; QOL based on EQ-5D-3L	EE based on 4-year follow-up RCT (Ising, 2017)	No	PSA	PsyMod
Suicide (*n* = 12)											
Lebenbaum (2020) [59] Canada	CUA (QALY) Societal	Suicide prevention campaigns *No intervention*	Markov	50 years *(1 year)*	Prior EE model and face validation with expert	Face validation (two psychiatrists)	Suicide rate; suicide re-attempt rate	Longitudinal data from 21 OECD countries; meta-analysis	Effect remains 1 year	Univariate, PSA	TreeAge Pro 2016
Kinchin (2020) [64] Australia	ROI (Monetary) Societal	School-based gatekeeper training (SafeTALK) *Status quo*	Markov	5 years *(3 months)*	Unclear	Not mentioned	RR reduction of hospitalized self-harm	Meta-analysis (for similar prevention, Sign of Suicide, 3 RCTs)	Effect remains 1 year	Univariate	Excel
Denchev (2018) [31] US	CEA (LY) Unclear perspective	Emergency Department-initiated interventions to reduce suicide risk *Usual care*	Markov	54 weeks *(6 weeks)*	RCT, expert opinion (but not detailed)	Not mentioned	Rate of suicide re-attempt	Similar RCTs (up to 5-year follow up)	Effect remains 3 months	Univariate, PSA	TreeAge Pro 15.2.1.0
Pil (2013) [60] Belgium	CUA (QALY) Societal	Suicide helpline *No intervention*	Markov	10 years *(1 year)*	Unclear	Not mentioned	Self-reported intend to die (before and after the call)	A pre-post intervention study	No	Univariate, PSA	Unclear
Comans (2013) [61] Australia	CUA (QALY) Societal	24-h crisis response telephone service *Usual care*	Markov	1 year	Bonanno’s model of grieving events	Not mentioned	Resilient and grieving	Bonanno’s model of grieving events (1 year follow up)	No	Univariate, PSA	Treeage 2011
Vasiliadis (2015) [32] Canada	CEA (LY) Societal	Multimodal suicidal prevention program *No program*	Unclear	Lifetime	Unclear	Not mentioned	Suicide attempt and suicide	RCT (NAD)	No	Univariate	Unclear
Godoy (2018) [62] US	CBA (monetary) Health sector	Anti-suicide multicomponent program *Do nothing*	Unclear	3 years	Unclear	Not mentioned	Suicide attempt	Repeated national survey on drug use and health	No	Univariate	Unclear
Damerow (2020) [29] SriLanka	CEA (LY) Health, other sectors	Anti-suicide gatekeeper training *No intervention*	Unclear	3 years	RCTs	Yes, only on time horizon, outcome	Fatal pesticide self-poisoning case	NA	No	Univariate	Excel
Atkins (2013) [63] US, Societal	CUA (DALY) Societal	Suicide barrier on the Golden Gate bridge *No intervention*	Unclear	20 years	Unclear	Not mentioned	Mortality reduction	San Francisco and Golden Gate Bridge suicides data	No	None	Unclear
Richardson (2017) [65] US	ROI (monetary) Payer	Postdischarge follow-up calls *No intervention*	Unclear	30 days	Unclear	Not mentioned	Readmission rate	RCT identified by a review (Luxton et al.)	No	Univariate, PSA	Unclear
Lee (2020) [30] 14 countries	CUA (HLYG) Health sector	Banning highly hazardous pesticides *Null comparator*	Markov	Lifetime (*1 year)*	WHO-Choice	Face validation (international expert panel)	Suicide mortality	Systematic review	Effect decreases over 5 years and remains from year 5 to lifetime (65%)	Univariate, PSA	Excel
Martínez-Alés (2021) [38] Spain	CEA (suicide attempt) Societal	Post-discharge suicide prevention *Treatment as usual*	Decision tree	1 year	Not mentioned	Not mentioned	Suicide re-attempt	RCT	No	Univariate, PSA	Excel
Bullying (*n* = 4)											
Persson (2018) [34] Sweden	CEA, CUA (QALY, victim free) Payer	School-based anti-bullying program (KiVa) *Treatment as usual*	Markov	9 years *(1 year)*	Unclear	Not mentioned	Bullying prevalence	Systematic review (cohort studies, up to 5-year follow up studies)	Intervention run over time horizon	Univariate, PSA	Unclear
Beckman (2015) [35] Sweden	CEA (victim free year) Payer	School-based anti-bullying program (Olweus) *No program*	Decision tree	3 years	Unclear	Not mentioned	Self-report of bully problems (2–3 times a month or more often)	Systematic review (cohort studies, up to 5-year follow-up studies)	intervention run over time horizon	Univariate, PSA	TreeAge Pro 2014
Huitsing (2019) [66] Netherlands	CBA (monetary) Societal	School-based anti-bullying program (Kiva) *No intervention*	Unclear	Lifetime	Unclear	Not mentioned	Self-report of bully problems	RCT (Kiva, 3-year follow-up)	Effects remain 70% in the long term	Univariate	Unclear
Hummel (2009) [28] UK	CUA (QALY) Unclear perspective	Anti-bullying program *No intervention*	Unclear	Lifetime	Literature review	Not mentioned	Bullying behaviour prevalence (bully, victim, bystander)	Evers et al., 2007	Effect remains lifetime	Univariate, PSA	Unclear
Violence (*n* = 4)											
Barbosa (2018) [69] UK	CUA (QALY) Societal	Identification and referral to improve safety (IRIS) *Usual care*	Markov	10 years *(6 months)*	Prior EE model	Not mentioned	Abuse identified; abuse event measured by Composite Abuse Scale (CAS)	RCTs (IRIS, MOSAIC)	Intervention runs over time horizon	Univariate, PSA	Unclear
Devine (2012) [67] UK	CUA (QALY) Societal	Identification and referral to improve safety (IRIS) *No program*	Markov	10 years *(6 months)*	Prior EE model on prevention of domestic violence (PreDoVe)	Not mentioned	Abuse identified	Prior EE model on prevention of domestic violence (PreDoVe)	Intervention run over time horizon	PSA	Unclear
Mallender (2013) [43] UK	CUA (QALY) Payer	Independence domestic violence advocacy services *No program*	Decision tree	3 months	Systematic review	Face validation (FGD, 6 times)	Domestic violence prevalence	A pre-post intervention study	No	Univariate	Excel
Norman (2010) [68] UK	CUA (QALY) Societal	System-based program for better detection and care for intimate partner violence (PreDoVe) *No program*	Markov	10 years (*6 months)*	Unclear	Not mentioned	abuse identified	RCT (PreDoVe)	Intervention runs over time horizon	Univariate	Unclear
Abuse (*n* = 3)											
Peterson (2018) [71] US	CBA (monetary) Societal	Early education intervention, providing services for a low-income family *No program*	Unclear	10 years *(unclear)*	Unclear	Not mentioned	CAN incidence	RCT (15-year follow-up); Chicago Longitudinal Study	No	Univariate	Excel
Dopp (2018) [70] US	CBA (monetary) Societal	Multisystemic Therapy for Child Abuse and Neglect *Standard outpatient services*	Unclear	Lifetime	Prior CBA model (WSIPP)	Not mentioned	Incidence of maltreatment and out-of-home replace measured by Conflict Tactics Scale (CTS)	RCT (Swenson et al., 2010)	Effect remains lifetime	Univariate, PSA	Excel
Kuklinski (2020) [72] US	CBA (monetary) Societal	Home visiting interventions *Referral calls*	Unclear	Lifetime	Prior CBA model (WSIPP)	Not mentioned	Out of home replacement and CANC incidence	RCT (the supportive parents project, SPP)	Effect remains lifetime	PSA	Unclear

Less than half of the included studies (*n* = 23) provided sufficient explanation for selecting the structure of the decision-analytic model. Only five studies were informed by systematic reviews [41, 43, 44] or literature reviews [28, 33]. Other five studies stated that the models were based on intervention clinical evidence (e.g., RCTs) [29, 31, 55], a disease classification [25] or evidence from cohort data [47]. The remaining 13 studies stated that the models were built based on previous models [30, 45, 49, 50, 52, 56, 59, 61, 67, 69, 70, 72, 73]. It is also worth noting that none of the included studies mentioned any competing theories regarding model structure.

Several structural assumptions were made for the purpose of modelling. The key assumptions included efficacy of interventions over a long term period, assumptions to simplify the model structure, assumptions relating to transition probabilities and treatment pathway, etc. To extrapolate the long-term intervention effectiveness, 29 studies assumed the intervention effect lasted over time. Of 29 studies, almost all did not mention whether these assumptions were validated. The authors often assumed that the intervention effect remained over time (i.e., for one year [40, 45, 47, 48, 59, 64], two years [73], four years [52], five years [30] or even a lifetime [28, 56, 70, 72]. They also assumed that the intervention effect gradually decreased with a specified decay rate. A decay rate of 50% was commonly used in included studies [46, 52, 58, 73]. Another common assumption to extrapolate the long term intervention effectiveness was that considering the interventions run over the time horizon [33–35, 50, 67–69].

However, the above structural assumptions, and the model structure in general, were rarely validated. In only eight models, expert opinions were stated to be used to conduct face validation [25, 30, 43, 59] or to provide justification on interventions [33, 45, 47] and time horizon [29]. Even in the mentioned models, the authors often provided little explanation [25, 33, 43, 45, 59] or no explanation [29, 30, 47] for the methods of employing experts in providing justifications for the model.

Although almost all studies evaluated all feasible and practical options relating to the stated decision problem, only 12 models provided detailed justification and criteria for excluding feasible options [25, 31, 35, 43, 45–47, 52, 54, 66, 71, 73].

The model's time horizon was considered sufficient to reflect all important differences between options in 30 studies (61.2%). Only ten models used a 50-year time horizon [50, 59] or lifetime horizon [27, 28, 30, 32, 56, 66, 70, 72]. In models with a shorter time horizon, only 22 studies (44.9%) justified the use of a shorter time horizon. In 27 Markov models, three studies (accounted for 11.0% of all Markov models) did not explicitly state the cycle length [47, 50, 52] and 11 studies (accounted for 40.7% of all Markov models) did not provide any justification for the chosen cycle length [31, 34, 44–46, 48, 57, 58, 60, 61, 73].

#### Data

Generally, methods for identifying data were evaluated as transparent and appropriate in all included studies. However, only 25 studies (51.0%) stated to use a systematic review to inform the selection of key parameters. For example, in terms of measuring intervention effect, 16 studies (32.7%) employed systematic review to identify intervention effect [27, 30, 34–37, 41, 44, 45, 47, 52, 55, 56, 59, 64, 73]. Meanwhile, 26 studies (53.1%) used evidence from a single trial. Other remaining studies identified key parameters of intervention effect from surveys [33, 62], longitudinal data [63] or pre-post intervention study [43, 49, 60].

In 13 studies, expert opinions were stated to be used to estimate particular parameter [29–31, 41, 42, 45, 47, 52, 55, 56, 64, 66]. Although the remaining studies did not report the use of expert opinion, they employed many authors’ own opinions in parameter estimations [26–28, 31, 32, 43, 65, 71]. Besides, it is worth noting that only four out of 13 studies that stated the use of expert opinions described the methods of getting expert opinions [25, 30, 45, 47].

Relating half-cycle correction, only six studies applied [25, 44, 59, 61, 64, 69]. The remaining models did not state the application of half-cycle correction and the reasons for the omission.

Regarding uncertainty assessment, three studies [36, 49, 63] did not perform any kind of uncertainty assessment. Only nine studies [26, 30, 41, 44–46, 58, 60, 73] performed all four principle types of uncertainty assessment (i.e., parameter uncertainty, structure uncertainty, methodology uncertainty and heterogeneity). Heterogeneity was the most common type of uncertainty being omitted (*n* = 40), followed by methodology uncertainty (*n* = 17) and structural uncertainty (*n* = 16).

Among 46 models that performed parameter uncertainty analysis, 12 studies only addressed univariate sensitivity analysis [26, 29, 32, 39, 40, 43, 56, 62, 64, 66, 68, 71]. Nine studies only performed probabilistic sensitivity analysis [25, 41, 44, 47, 48, 54, 55, 67, 72]. The remaining 26 studies performed both univariate sensitivity analysis and probabilistic sensitivity analysis. Although it is recommended that the ranges used for sensitivity analysis be stated clearly and justified, many models did not specify the value ranges and their reasons [36, 39, 40, 49, 54, 55, 57, 58, 60, 61, 63, 71, 72]. Besides, only 12 studies clearly described and justified the choice of distribution for each parameter [25, 30, 33, 35, 37, 38, 42, 47, 50, 53, 57, 67].

#### Consistency

There was limited evidence that the mathematical logic of the models in included studies had been tested thoroughly before use. Only one study [25] mentioned that the model was validated based on the Assessment of the Validation Status of Health Economics decision models (AdViSHe) questionnaire [74]. Indeed, the mathematical logic of the model was validated by extreme value testing and by checking whether the relative number of patients in each cycle and state was consistent with empirical evidence [25].

Only six studies [25, 56, 64, 67–69] (12.2%) mentioned the application of model calibration for transition probabilities [25, 64, 67–69], epidemiological outcomes [25] and cost outcomes [56].

More than half of the studies (*n* = 29, 59.2%) compared their results with other models’ results and explained the reasons for any differences. The remaining 20 studies did not mention any earlier models for reference.

### Cost-effectiveness

As mentioned in the analysis method, we used the dominance ranking metrics for the qualitative synthesis of the cost-effectiveness results of included studies (See Table 5). More detailed information on the cost-effectiveness of included studies could be found in Online Supplementary File (Table S4).

**Table 5:** The dominance ranking matrix

Incremental cost	Incremental outcome	Authors	Intervention	Comparator	Horizon	Outcome	ICER (in 2020 US$ value)	Cos-effective?
Depression								
+	+	Lee (2017) [45]	Group-based psychological intervention (Universal)	No intervention	10 years	DALY	AU$ 7,350/DALY (5,645)	Yes
+	+	Lee (2017) [45]	Group-based psychological intervention (Indicated)	No intervention	10 years	DALY	AU$19,550/DALY (15,015)	Yes
+	+	Mihalopoulos (2011) [7]	Opportunistic screening for Sub-syndromal depression + brief bibliotherapy	Do-nothing	5 years	DALY	AU$8,600 (9,303)	Yes
+	+	Mihalopoulos (2011) [7]	Opportunistic screening for Sub-syndromal depression + psychological group ther apy	Do-nothing	5 years	DALY	AU$20,000 (21,635)	Yes
+	+	Paulden (2010) [41]	Routine screening for postnatal depression + psychological therapy	Usual care	1 year	QALY	Lowest ICER £41,103/QALY (74,419)	No
+	+	Hunter (2014) [44]	Screening with a Risk Algorithm (PredictD) + low-intensity prevention program	Treatment as usual	1 year	QALY	£9,607/QALY (16,603)	Yes
+	+	Hunter (2014) [44]	Universal screening + low-intensity prevention program	Treatment as usual	1 year	QALY	£83,356/QALY (142,900)	No
+	+	Lokkerbol (2014) [47]	Preventive telemedicine (remain curative care coverage)	Usual care	5 years	DALY, monetary	ROI = 1.76	Yes
0	+	Lokkerbol (2014) [47]	Preventive telemedicine (reduce curative care coverage)	Usual care	5 years	DALY, monetary	ROI = 1.77	Cost-saving
+	+	Mihalopoulos (2012) [7]	screening + psychological intervention	Do-nothing	5 years	DALY	AU$5400 (5,841)	Yes
+ (healthcare) - (societal)	+	van den Berg (2011) [48]	Opportunistic screening + minimal contact psychotherapy	Usual care	5 years	DALY	€1,400 (healthcare); cost-saving (societal)	Yes
-	+	Ssegonja (2020) [37]	Group-based CBT	No intervention	5,10 years	QALY, cases	Dominant	Cost-saving
+	+	Valenstein (2001) [27]	Depression Screening	No intervention	Lifetime	QALY	US$225,467/QALY (payer); 192,444/QALY (societal)	No
+	+	Lintvedt OK (2013) [40]	e-CBT	No intervention	1 year	QALY	NOK$ 3,432/QALY (505)	Yes
+	+	Jiao (2017) [50]	Depression screening (PHQ-2, PHQ-9) + collaborative care	Usual care	50 years	QALY	US$1,726/QALY (1,979)	Yes
-	+	Goetzel (2014) [49]	Workplace health risk management program	No intervention	1 year	Monetary	ROI = 2.03	Cost-saving
+	+	Premji (2021) [42]	Screening for depression and follow-up diagnosis and treatment	No screening	2 years	QALY	US$ 17,644 (18,012)	No
-	+	Feldman (2020) [51]	Group-based cognitive behaviour therapy	No intervention	5,10 years	QALY	Dominant	Cost-saving
Eating Disorder								
+	+	Le (2017) [52]	Cognitive dissonance intervention	No intervention	≥ 10 years	DALY	AU$ 103,980/DALY (70,862)	No
-	+	Kass (2017) [36]	Screening + online preventive or treatment	Wait list control	< 5 years	Cases	Dominant	Cost-saving
-	+	Wang (2011) [53]	School-based education and physical activity (Planet Health)	Usual curricula	≥ 10 years	QALY	Dominant	Cost-saving
+	+	Wright (2014) [33]	School-based eating disorder screening	No intervention	≥ 10 years	QALY, LY with ED	US$ 9,041/LY with ED avoided (10,369) US$ 56,500/QALYs (64,800)	Yes
Anxiety								
-	+	Ophuis (2018) [55]	CBT-based early intervention for subthreshold panic disorder	Usual care	5–9 years	QALY	Dominant	Cost-saving
+	+	Mihalopoulos (2015) [54]	Screening and parenting educational program	Do-nothing	< 5 years	DALY	AU$ 8,000/DALY ($6,144)	Yes
+	+	Simon (2013) [39]	Screening + early child/parental focused intervention	Do-nothing	< 5 years	Cases	€107/AIDS improved child ($13.88)	Yes
-	+	Kumar (2018) [56]	Mobile CBT	No/traditional CBT	≥ 10 years	QALY	Dominant	Cost-saving
+	+	Richardson (2017) [65]	Post-discharge follow-up calls	Do-nothing	< 5 years	monetary	ROI = 1.76 (commercial); ROI = 2,05 (Medicaid)	Yes
Behavior Disorder								
+ (Nystrand, 2019) [58] - (Nystrand, 2020) [57]	+	Nystrand (2019, 2020) [57, 58]	Group-based indicated parenting programs (Comet)	Wait list control	til 18–20 years old	DALY	US$ 972/DALY (1,172)	Yes
-	+	Nystrand (2019, 2020) [57, 58]	Group-based indicated parenting programs (Connect)	Wait list control	til 18–20 years old	DALY	Dominant	Cost-saving
+ (Nystrand, 2019) [58] - (Nystrand, 2020) [57]	+	Nystrand (2019, 2020) [57, 58]	Group-based indicated parenting programs (IY)	Wait list control	til 18–20 years old	DALY	US$224/DALY (354)	Yes
-	+	Nystrand (2019, 2020) [57, 58]	Group-based indicated parenting programs (COPE)	Wait list control	til 18–20 years old	DALY	Dominant	Cost-saving
-	+	Nystrand (2019, 2020) [57, 58]	Group-based indicated parenting programs (Bibliotherapy)	Wait list control	til 18–20 years old	DALY	Dominant	Cost-saving
-	+	Mihalopoulos, C., et al [26]	Multi-level system of parenting and family support (Triple P)	No intervention	26 years	Cases	Dominant	Cost-saving
Psychosis								
-	+	Wijnen (2020) [25]	CBT-based intervention for Ultra-high risk	Usual care	10 years	QALY	Dominant	Cost-saving
Suicide								
+	+	Lebenbaum (2020) [59]	Suicide prevention campaigns	No intervention	50 years	QALY	CAD$ 18,853/QALY (16,916)	Yes
- (Mackay) + (Others)	+	Kinchin (2020) [64]	School-based gatekeeper training (SafeTALK)	Status quo	5 years	Monetary	ROI = 31.2 (Mackay) 4.1 (Queensland) 3.3 (Australia)	Yes
-	+	Denchev (2018) [31]	Emergency Department-initiated interventions to reduce suicide risk (Postcard)	Usual care	54 weeks	LY	Dominant	Cost-saving
+	+	Denchev (2018) [31]	Emergency Department-initiated interventions to reduce suicide risk (Telephone)	Usual care	54 weeks	LY	US$ 4,300/LY (4,756)	Yes
+	+	Denchev (2018) [31]	Emergency Department-initiated interventions to reduce suicide risk (CBT)	Usual care	54 weeks	LY	US$ 18,800/LY (20,796)	Yes
-	+	Pil (2013) [60]	Suicide helpline	No intervention	10 years	QALY	Dominant	Cost-saving
-	+	Comans (2013) [61]	24-h crisis response telephone service	Usual care	1 year, 5 years	QALY	Dominant	Cost-saving
+	+	Vasiliadis (2015) [32]	Multimodal suicidal prevention program	No intervention	Lifetime	LY	CAD$ 3,979/LY (3,863)	Yes
-	+	Godoy (2018) [62]	Anti-suicide multicomponent program	Do-nothing	3 years	Monetary	BCR = 4.5	Cost-saving
+	+	Damerow (2020) [29]	Anti-suicide gatekeeper training	No intervention	3 years	LY	0.23 fatal cases needed to be prevented to be cost-effectiveness	Yes
+	+	Atkins (2013) [63]	Suicide barrier on the Golden Gate bridge	No intervention	20 years	DALY	US$ 4,876/DALY (5,818)	Yes
+	+	Lee (2020) [30]	Banning highly hazardous pesticides	Null comparator	Lifetime	HLYGs	Lower income setting: $I94/HLYG; Higher income setting: $I237/HLYG	Yes
+	+	Martínez-Alés (2021) [38]	Post-discharge suicide prevention strategies based on Enhanced Contact	Treatment as usual	1 year	Suicide attempt averted	€2340 (3,119)	Yes
+	+	Martínez-Alés (2021) [38]	Post-discharge suicide prevention strategies based on Psychotherapy	Treatment as usual	1 year	Suicide attempt averted	€6260 (8,345)	Yes
Bullying								
+	+	Persson (2018) [34]	School-based anti-bullying program (KiVa)	Treatment as usual	9 years	QALY, victim-free	SEK 13,1321/QALY (18,812) SEK 7,879/victim-free year (1,128)	No
+	+	Beckman (2015) [35]	School-based anti-bullying program (Olweus)	No intervention	3 years	Victim-free year	SEK 131,250/victim free year (18,801)	Yes
+	+	Huitsing (2019) [66]	School-based anti-bullying program (Kiva)	No intervention	Lifetime	Monetary	ROI = 4.04 to 6.72	Yes
+	+	Hummel (2009) [28]	Anti-bullying program	No intervention	Lifetime	QALY	£9,600/QALY (18,345)	Yes
Violence								
-	+	Barbosa (2018) [69]	Identification and referral to improve safety (IRIS)	Usual care	10 years	QALY	Dominant	Cost-saving
-	+	Devine (2012) [67]	Identification and referral to improve safety (IRIS)	No intervention	10 years	QALY	Dominant	Cost-saving
-	+	Mallender (2013) [43]	Independence domestic violence advocacy services	No intervention	3 months	QALY	Dominant	Cost-saving
+	+	Norman (2010) [68]	System-based program for better detection and care for women experiencing intimate partner violence (PreDoVe)	No intervention	10 years	QALY	£742/QALY (1,417)	Yes
Abuse								
+ (payer) - (societal)	+	Peterson (2018) [71]	Early education intervention for low-income families (Child-parent Centers model, preschool only)	No intervention	10 years	Monetary	BCR = 0.53 (payer) BCR = 1.73 (societal)	Yes
+ (payer) - (societal)	+	Peterson(2018) [71]	Early education intervention for low-income families (Child-parent Centers model, Preschool and School-age)	No intervention	10 years	Monetary	BCR = 0.55 (payer) BCR = 1.80 (societal)	Yes
-	+	Peterson (2018) [71]	Early education intervention for low-income families (Nurse-family partnership model)	No intervention	10 years	Monetary	BCR = 1.79 (payer) BCR = 6.3 (societal)	Cost-saving
+	+	Dopp (2018) [70]	Multisystemic Therapy for Child Abuse and Neglect	Usual care	Lifetime	Monetary	BRC = 3.31	Yes
+	+	Kuklinski (2020) [72]	Home visiting intervention	Referral calls	Lifetime	Monetary	BCR = 5.19 to 19.05	Yes

Among 61 interventions that were analyzed in 49 included studies, no intervention was dominated (i.e., less effective but more costly). Twenty-one interventions (34.4% of interventions) were classified as “favour” because they were more effective but less costly. Most of them were selective or indicated prevention interventions (17 out of 21 interventions), were modelled from a time horizon of five years and above (14 out of 21 interventions), were targeted for the prevention of depression (*n* = 4), behavioural disorder (*n* = 4), suicide (*n* = 4), violence (*n* = 3), anxiety (*n* = 2), eating disorder (*n* = 2), abuse (*n* = 1), and psychosis (*n* = 1).

The remaining 40 interventions (65.6%) delivered better health outcomes but at a higher cost. Based on the authors’ conclusions and the thresholds provided, almost all of them (34 out of 40 interventions) were “value for money”, given that the ICER remained under corresponding thresholds (typically US$50,000 – US$100,000 in the US, AU$50,000 in Australia, £20,000-£30,000 in the UK) or ROI was greater than 1. Only six interventions, which four prevented depression in the adult population [27, 41, 42, 44], one intervention focused on eating disorders [52], and one intervention that prevented bullying in the children and adolescent population [34] were considered to be not cost-effective since the ICERs were above the thresholds.

## Discussion

This systematic review has shown the current situation in published decision-analytic models for mental health prevention interventions. Although there were similar systematic reviews on economic evaluations of mental health prevention interventions, they did not focus on model-based studies. Thus, this systematic review is the first to try to summarise and critically appraise all model-based economic evaluations in the field. The results of this review will provide more evidence to support practice and policy with evidence on medium and long term cost-effectiveness of mental health prevention and aid researchers in improving the quality of future decision-analytic models.

There has been a rapid increase in the number of economic evaluation models in this field, with more than half of included models being published in the last five years (i.e., 2015 to 2020). However, almost all included models were conducted for higher-income countries rather than lower-income countries despite the fact that the burden of mental health problems (in terms of DALYs) is increasing more rapidly in lower-income countries than in their higher-income counterparts [75]. The most common type of economic evaluation was CUA, with the dominant use of QALY as the primary outcome and the application of the Markov model from the societal or health sector perspective. A wide range of prevention strategies was evaluated in the included studies, with the dominance of selective or indicated prevention. It is easy to understand since universal prevention intervention is believed to be more costly than its alternatives. Interventions in included studies also targeted a wide range of mental health problems and risk factors, in which interventions targeted depression and suicide were dominant. This review calls for more decision-analytic models in the future that diversify the topic of mental health problems being addressed, the type of prevention strategies (that focus more on universal prevention intervention) being evaluated and the context of intervention (that focus more on lower-income countries).

Despite a high level of heterogeneity relating to study scope and model structure among included decision-analytic models, almost all mental health prevention interventions were cost-saving (21 interventions, accounting for 34.4%) or cost-effective (34 interventions, accounting for 55.7%). This review identified a large number of interventions for mental health prevention that are cost-saving. All cost-saving interventions have characteristics of indicated or selective prevention strategies, except for one anti-suicide multicomponent program (which had a universal component along with indicated and selective component) [62]. The target population in the cost-saving interventions were often adults (80.9% of cost-saving interventions). They also tended to be analyzed in a longer time horizon (i.e., 12 out of 21 cost-saving interventions were captured in a time horizon of ten years or more). None of the included interventions was less effective but more costly. It is different from the findings of a similar review [9], in which two interventions on depression prevention (which were assessed in a trial-based economic evaluation) were less effective but more costly.

### Quality of decision-analytic models

Critically appraising the quality of the included studies revealed several significant limitations of included decision-analytic models. Firstly, a large number of papers reported little or no details of the model structures and the rationale for choosing the models. Only in five studies, the model structures were informed by the systematic reviews or literature reviews. Secondly, although one of the advantages of applying modelling is that it allows estimating interventions’ cost and outcome over a sufficient time horizon outside RCTs, many included models in this review were only modelled for one year or less. Thirdly, the structural assumptions, notably those assumptions needed to extrapolate the short-term outcome of intervention into long-term outcome, were rarely validated. Even in the studies that mentioned the use of expert opinions to validate the assumptions, the report of the method used was insufficient. Fourthly, systematic reviews were not used to identify the key parameters such as intervention effect in many included studies. Fifthly, there was limited evidence that the mathematical logic of the models in included studies had been tested thoroughly before use. Internal validation techniques such as extreme value testing or model calibration were only mentioned in a minimal number of studies. Sixthly, many studies skipped performing at least one in four principal types of uncertainty analysis, i.e., parameter uncertainty, structure uncertainty, methodology uncertainty and heterogeneity. Notably, three studies did not perform any kind of uncertainty analysis despite the crucial role of uncertainty analysis in modelling studies. Lastly, many studies remained to be lack details and transparency in reporting their model structures (e.g., specified primary decision-makers, perspectives) and in the data selection/incorporation process (e.g., quality of data, justification for the choice of distribution, reason for the omission of half-cycle correction).

 This review also calls for future decision-analytic models to improve their quality to better inform the policy-making process. The model structure should be sufficiently described, and evidence to inform the model structure should also be better provided. Similar to recommendations by other authors [3, 9], our review continues to call for the application of a longer time horizon to fully capture the costs and outcomes of mental health prevention interventions. To do so, the structural assumptions, notably those assumptions needed to extrapolate the short-term outcomes of intervention into long-term outcomes, were inevitable and necessary to be better reported and validated. Authors of future models should make efforts to validate the model, especially for model structure, model assumptions, and the mathematical logic of the models. Authors might consult the Assessment of the Validation Status of Health-Economic decision models (AdViSHe) questionnaire for this purpose [74]. Other methodological limitations should also be improved, such as applying a more systematic method for identifying key model parameters, addressing not only parameter uncertainty but also structure uncertainty, methodology uncertainty and heterogeneity. The quality of the reporting decision-analytic model should also be improved by applying a guideline or checklist specialised in modelling techniques, such as the Philips checklist [20] or the ISPOR checklist [76].

### Strengths and limitations

This review is the first to focus on model-based economic evaluations of mental health prevention. Previous systematic reviews [9, 77, 78] commonly addressed trial-based economic evaluation studies, examined short-term costs and consequences and did not reflect real-life practice. Thus, our search strategy was more sensitive in detecting model-based economic evaluations. Our review comprehensively covers a wide range of mental health problems and well-known related issues such as suicide, violence, bullying or abuse. We also did not apply any restriction on beneficences age, economic evaluation type and publication year. Our review also critically appraised the quality of the included studies by the Philips Checklist, which is recommended for addressing model-based economic evaluations.

Our review has some limitations. Firstly, our search strategy only used English keywords to search for relevant records from proposed electronic databases and other sources. The study selection also included only records that their full texts were available in English. Thus, potentially relevant studies could be missed. Secondly, since many studies did not have a clear model structure, it was challenging to apply some items of the Philips Checklist, for example, the appraisal items related to transition probabilities or cycle length. Lastly, a wide range of mental health issues was covered in our review. We excluded studies that could not distinguish between mental health outcomes and other outcomes, e.g. physical outcomes, educational outcomes, and development outcomes. Besides, although it was not initially suggested to quantify the responses to the Philips Checklist, we applied a scoring approach to estimate the percentage of items fulfilled. By doing so, we must assume equal weighting to all criteria, even though some criteria might be more critical than others.

## Conclusions

This review is the first to focus on decision-analytic models for mental health prevention. There is an increasing number of decision-analytic models. Still, evidence has limited to higher-income countries, in the most common mental health problems (e.g., depression and suicide), and still limited to the short-term horizon. Despite a high level of heterogeneity relating to study scope and model structure among included decision-analytic models, almost all mental health prevention interventions were cost-saving or cost-effective to invest in. Researchers should develop more models in the low-resource context, expand the time horizon, improve the evidence identification to inform model structure and inputs, and improve the practice of model validation.

## Supplementary Information

**Additional file 1: Table S1.** The PRISMA 2020 Checklist. **Table S2.** Search Strategy. **Table S3.** Reasons for Fultext Exclusion. **Table S4.** Summary of cost-effectiveness results in the included studies.

## Data Availability

All data generated or analysed during this study are included in this published article [and its supplementary information files].
