# Supplementary material for: Modelling in economic evaluation of mental health prevention: current status and quality of studies
Source: BMC Health Serv Res. 2022 Jul 13;22:906. doi: 10.1186/s12913-022-08206-9 (PMC9281039; doi:10.1186/s12913-022-08206-9)
Supplement: Supplementary file 1 — Additional file 1: Table S1. The PRISMA 2020 Checklist. Table S2. Search Strategy. Table S3. Reasons for Fultext Exclusion. Table S4. Summary of cost-effectiveness results in the included studies. [file 12913_2022_8206_MOESM1_ESM.docx]

**ONLINE SUPPLEMENTARY FILE**

**Modelling in Economic Evaluation of mental health prevention: Current status and quality of studies**

Nguyen Thu Ha^1^, Nguyen Thanh Huong^2*^, Vu Nguyen Anh^3^, Nguyen Quynh Anh^1^

^1^Department of Health Policy and Economics, Hanoi University of Public Health, Hanoi, Vietnam

^2^ Department of Health Education and Promotion, Hanoi University of Public Health, Hanoi, Vietnam

^3^ Bachelor Student, Hanoi University of Public Health, Hanoi, Vietnam

* Corresponding author:

Email: [nth@huph.edu.vn](mailto:nth@huph.edu.vn)

**Table S1. The PRISMA 2020 Checklist**

| **Section and Topic** | **Item #** | **Checklist item** | **Location where item is reported** |
| --- | --- | --- | --- |
| **TITLE** | | |  |
| Title | 1 | Identify the report as a systematic review. | Title (p.1) |
| **ABSTRACT** | | |  |
| Abstract | 2 | See the PRISMA 2020 for Abstracts checklist. | Abstract (p.2) |
| **INTRODUCTION** | | |  |
| Rationale | 3 | Describe the rationale for the review in the context of existing knowledge. | Introduction (p.3) |
| Objectives | 4 | Provide an explicit statement of the objective(s) or question(s) the review addresses. | Introduction (p.3) |
| **METHODS** | | |  |
| Eligibility criteria | 5 | Specify the inclusion and exclusion criteria for the review and how studies were grouped for the syntheses. | Method (p.3-4)  Table 1 |
| Information sources | 6 | Specify all databases, registers, websites, organisations, reference lists and other sources searched or consulted to identify studies. Specify the date when each source was last searched or consulted. | Method (p.4) |
| Search strategy | 7 | Present the full search strategies for all databases, registers and websites, including any filters and limits used. | Sup Table S.2 |
| Selection process | 8 | Specify the methods used to decide whether a study met the inclusion criteria of the review, including how many reviewers screened each record and each report retrieved, whether they worked independently, and if applicable, details of automation tools used in the process. | Selection process (p.4) |
| Data collection process | 9 | Specify the methods used to collect data from reports, including how many reviewers collected data from each report, whether they worked independently, any processes for obtaining or confirming data from study investigators, and if applicable, details of automation tools used in the process. | Data extraction (p.4) |
| Data items | 10a | List and define all outcomes for which data were sought. Specify whether all results that were compatible with each outcome domain in each study were sought (e.g. for all measures, time points, analyses), and if not, the methods used to decide which results to collect. | Data extraction (p.4) |
|  | 10b | List and define all other variables for which data were sought (e.g. participant and intervention characteristics, funding sources). Describe any assumptions made about any missing or unclear information. | Data extraction (p.4) |
| Study risk of bias assessment | 11 | Specify the methods used to assess risk of bias in the included studies, including details of the tool(s) used, how many reviewers assessed each study and whether they worked independently, and if applicable, details of automation tools used in the process. | Quality assessment (p.4-5) |
| Effect measures | 12 | Specify for each outcome the effect measure(s) (e.g. risk ratio, mean difference) used in the synthesis or presentation of results. | NA |
| Synthesis methods | 13a | Describe the processes used to decide which studies were eligible for each synthesis (e.g. tabulating the study intervention characteristics and comparing against the planned groups for each synthesis (item #5)). | Data synthesis (p.5) |
|  | 13b | Describe any methods required to prepare the data for presentation or synthesis, such as handling of missing summary statistics, or data conversions. |  |
|  | 13c | Describe any methods used to tabulate or visually display results of individual studies and syntheses. |  |
|  | 13d | Describe any methods used to synthesize results and provide a rationale for the choice(s). If meta-analysis was performed, describe the model(s), method(s) to identify the presence and extent of statistical heterogeneity, and software package(s) used. |  |
|  | 13e | Describe any methods used to explore possible causes of heterogeneity among study results (e.g. subgroup analysis, meta-regression). |  |
|  | 13f | Describe any sensitivity analyses conducted to assess robustness of the synthesized results. |  |
| Reporting bias assessment | 14 | Describe any methods used to assess risk of bias due to missing results in a synthesis (arising from reporting biases). | NA |
| Certainty assessment | 15 | Describe any methods used to assess certainty (or confidence) in the body of evidence for an outcome. | NA |
| **RESULTS** | | |  |
| Study selection | 16a | Describe the results of the search and selection process, from the number of records identified in the search to the number of studies included in the review, ideally using a flow diagram. | Results (p.5)  Figure 2 |
|  | 16b | Cite studies that might appear to meet the inclusion criteria, but which were excluded, and explain why they were excluded. | Results (p.5)  Sup Table S3 |
| Study characteristics | 17 | Cite each included study and present its characteristics. | Study characterisistic (p.5)  Table 2 |
| Risk of bias in studies | 18 | Present assessments of risk of bias for each included study. | Quality assessment (p.6-8)  Table 3 |
| Results of individual studies | 19 | For all outcomes, present, for each study: (a) summary statistics for each group (where appropriate) and (b) an effect estimate and its precision (e.g. confidence/credible interval), ideally using structured tables or plots. | Cost-effectiveness (p.8)  Sup Table S4 |
| Results of syntheses | 20a | For each synthesis, briefly summarise the characteristics and risk of bias among contributing studies. | Cost-effectiveness (p.8)  Sup Table S4 |
|  | 20b | Present results of all statistical syntheses conducted. If meta-analysis was done, present for each the summary estimate and its precision (e.g. confidence/credible interval) and measures of statistical heterogeneity. If comparing groups, describe the direction of the effect. |  |
|  | 20c | Present results of all investigations of possible causes of heterogeneity among study results. |  |
|  | 20d | Present results of all sensitivity analyses conducted to assess the robustness of the synthesized results. |  |
| Reporting biases | 21 | Present assessments of risk of bias due to missing results (arising from reporting biases) for each synthesis assessed. | NA |
| Certainty of evidence | 22 | Present assessments of certainty (or confidence) in the body of evidence for each outcome assessed. |  |
| **DISCUSSION** | | |  |
| Discussion | 23a | Provide a general interpretation of the results in the context of other evidence. | Discussion (p.8-10) |
|  | 23b | Discuss any limitations of the evidence included in the review. | Discussion (p.10) |
|  | 23c | Discuss any limitations of the review processes used. | Discussion (p.10) |
|  | 23d | Discuss implications of the results for practice, policy, and future research. | Discussion (p.10) |
| **OTHER INFORMATION** | | |  |
| Registration and protocol | 24a | Provide registration information for the review, including register name and registration number, or state that the review was not registered. | Abstract, Method (p. 3) |
|  | 24b | Indicate where the review protocol can be accessed, or state that a protocol was not prepared. | Method (p. 3) |
|  | 24c | Describe and explain any amendments to information provided at registration or in the protocol. |  |
| Support | 25 | Describe sources of financial or non-financial support for the review, and the role of the funders or sponsors in the review. | Declarations (p.11) |
| Competing interests | 26 | Declare any competing interests of review authors. | Declarations (p.11 |
| Availability of data, code and other materials | 27 | Report which of the following are publicly available and where they can be found: template data collection forms; data extracted from included studies; data used for all analyses; analytic code; any other materials used in the review. | Declarations (p.11 |

*From:*  Page MJ, McKenzie JE, Bossuyt PM, Boutron I, Hoffmann TC, Mulrow CD, et al. The PRISMA 2020 statement: an updated guideline for reporting systematic reviews. BMJ 2021;372:n71. doi: 10.1136/bmj.n71

**Table S2: Search Strategy**

Date of last perform: 11 Nov 2021

| **Name of database: PubMed** | |
| --- | --- |
| # | Seaching strategy |
| 1 | mental health[MeSH Terms] OR mental health[Title/Abstract] OR mental well being[Title/Abstract] OR mental wellbeing[Title/Abstract] OR mental well-being[Title/Abstract] OR psychologic* well being[Title/Abstract] OR psychologic* wellbeing[Title/Abstract] OR (psychologic* well-being[Title/Abstract] OR psychologic* problem*[Title/Abstract] OR psychologic* disorder*[Title/Abstract] OR (psychologic* disturbance*[Title/Abstract] OR mental disease*[Title/Abstract] OR mental disorder[MeSH Terms] OR (mental disorder*[Title/Abstract] OR mental illness*[Title/Abstract] OR psycho*[Title/Abstract] |
| 2 | aggression[MeSH Terms] OR aggression[Title/Abstract] OR aggressive*[Title/Abstract] OR anger[Title/Abstract] OR mood[Title/Abstract] OR emotion*[Title/Abstract] OR stress*[Title/Abstract] OR obsess*[Title/Abstract] OR anger[MeSH Terms] |
| 3 | antisocial personality disorder[MeSH Terms] OR antisocial disorder*[Title/Abstract] OR anti social disorder*[Title/Abstract] OR antisocial behavio*[Title/Abstract] OR anti social behavio*[Title/Abstract] OR anti social behavio*[Title/Abstract] OR anti-social disorder*[Title/Abstract] OR antisocial personality disorder*[Title/Abstract] OR anti social personality disorder*[Title/Abstract] OR anti social personality disorder*[Title/Abstract] |
| 4 | anxiety[MeSH Terms] OR depressive disorder[MeSH Terms] OR depression[MeSH Terms] OR conduct disorder[MeSH Terms] OR anxiety[Title/Abstract] OR depress*[Title/Abstract] OR conduct disorder*[Title/Abstract] OR behavio* disorder*[All Fields] OR behavio* problem*[Title/Abstract] |
| 5 | social alienation[MeSH Terms] OR social alienation*[Title/Abstract] OR impulsive[Title/Abstract] OR impulsivity[Title/Abstract] OR social isolation[Title/Abstract] OR isolation[Title/Abstract] |
| 6 | conscious*[Title/Abstract] OR selfesteem[Title/Abstract] OR self-esteem[Title/Abstract] OR self-esteem[Title/Abstract] OR self-efficacy[Title/Abstract] OR self-efficacy[Title/Abstract] OR selfefficacy[Title/Abstract] |
| 7 | suicide[MeSH Terms] OR physical abuse[MeSH Terms] OR child abuse[MeSH Terms] OR adverse childhood experiences[MeSH Terms] OR violence[MeSH Terms] OR suicide[Title/Abstract] OR bullying[Title/Abstract] OR bully[Title/Abstract] OR maltreatment[Title/Abstract] OR abuse[Title/Abstract] OR neglect*[Title/Abstract] OR violence[Title/Abstract] OR pressure[MeSH Terms] OR pressure[Title/Abstract] OR adverse childhood experienc*[Title/Abstract] OR ace[Title/Abstract] |
| 8 | OR #1-#7 |
| 9 | (health education[MeSH Terms]) OR (health promotion[MeSH Terms]) OR (promot*[Title/Abstract]) OR (prevent*[Title/Abstract]) OR (early intervent*[Title/Abstract]) OR (prevention[MeSH Subheading] AND control[MeSH Subheading]) |
| 10 | #8 AND #9 |
| 11 | (cost-benefit analysis[MeSH Terms]) OR (Quality-Adjusted Life Years[MeSH Terms])) OR (Cost and Cost Analysis[MeSH Terms])) OR (economic evaluation[Title/Abstract])) OR (economic analy*[Title/Abstract])) OR (health economic*[Title/Abstract])) OR (value for money[Title/Abstract])) OR (value-for-money[Title/Abstract])) OR (cost benefit*[Title/Abstract])) OR (cost-benefit*[Title/Abstract])) OR (cost efficien*[Title/Abstract])) OR (cost-efficien*[Title/Abstract])) OR (costefficien*[Title/Abstract])) OR (cost utilit*[Title/Abstract])) OR (cost-utilit*[Title/Abstract])) OR (costutilit*[Title/Abstract])) OR (cost minimi*[Title/Abstract])) OR (cost-minimi*[Title/Abstract])) OR (costminimi*[Title/Abstract])) OR (cost effective*[Title/Abstract])) OR (cost-effective*[Title/Abstract])) OR (costeffective*[Title/Abstract])) OR (cost evaluation[Title/Abstract])) OR (cost consequen*[Title/Abstract])) OR (cost identificat*[Title/Abstract])) OR (cost analy*[Title/Abstract])) OR (cost containment[Title/Abstract])) OR (cost stud*[Title/Abstract])) OR (quality adjusted life year*[Title/Abstract])) OR (quality-adjusted life year*[Title/Abstract])) OR (qualityadjusted life year*[Title/Abstract])) OR (disability adjusted life year*[Title/Abstract])) OR (disability-adjusted life year*[Title/Abstract])) OR (disabilityadjusted life year*[Title/Abstract])) OR (qaly*[Title/Abstract])) OR (daly*[Title/Abstract]) |
| 12 | (Markov Chain[MeSH Terms]) OR (Decision Trees[MeSH Terms])) OR (modeling[Title/Abstract])) OR (modelling[Title/Abstract])) OR (model* based[Title/Abstract])) OR (model-based[Title/Abstract])) OR (model* technique*[Title/Abstract])) OR (decision analy* model [Title/Abstract])) OR (markov model*[Title/Abstract])) OR (markov-model*[Title/Abstract])) OR (simulation model*[Title/Abstract])) OR (system dynamic model*[Title/Abstract])) OR (discrete event simulation*[Title/Abstract])) OR (discrete-event simulation*[Title/Abstract])) OR (microsimulation[Title/Abstract])) OR (micro-simulation[Title/Abstract])) OR (agent based model*[Title/Abstract])) OR (agent-based model*[Title/Abstract])) OR (cohort model*[Title/Abstract])) OR (decision tree*[Title/Abstract])) OR (probabilit*[Title/Abstract])) OR (transition[Title/Abstract])) OR (patient level simulation[Title/Abstract])) OR (patient-level simulation[Title/Abstract]) |
| 13 | #10 AND #11 AND #12 |
| 14 | Filters applied: Humans |

NB: MeSH: Medical Subject Headings

| **Name of database: Psycinfo (via Psycnet)** | |
| --- | --- |
| # | Searching strategies |
| 1 | "mental health" OR "mental well being" OR "mental wellbeing" OR "psychologic* well being" OR "psychologic* wellbeing" OR "psychologic* problem*" OR "psychologic* disorder*" OR "psychologic* disturbance*" OR "mental disease*" OR "mental disorder*" OR "mental illness*" OR "psycho*" |
| 2 | "aggression" OR "aggressive*"OR "anger" OR "mood" OR "emotion*" OR "stress*" OR "obsess*" |
| 3 | "antisocial disorder*" OR "anti social disorder*" OR "antisocial behavio*" OR "anti social behavio*" OR "antisocial personality disorder*" OR "anti social personality disorder*" |
| 4 | "anxiety" OR "depress*" OR "conduct disorder*" OR "behavio* disorder*" OR "behavio* problem*" |
| 5 | "social alienation" OR "impulsive*" OR "impulsivity" OR "isolation" |
| 6 | "conscious*" OR "selfesteeem" OR "self esteem" OR "self efficacy" OR "selfefficacy" |
| 7 | "suicide" OR"bully" OR "bullying" OR "maltreatment" OR "abuse" OR "neglect*" OR "violence" OR "pressure" OR "adverse childhood experienc*" OR "ACE*" |
| 8 | ti, ab, kw, it: OR #1-#7 |
| 9 | ALL: "promot*" OR "prevent*" OR "health education" OR "prevention and control" OR "early intervent*" |
| 10 | #8 AND #9 |
| 11 | ALL:"economic evaluation" OR "economic analy*" OR "health economic*" OR "value for money" OR "cost benefit*" OR "cost effective*" OR "cost efficien*" OR "cost utilit*" OR "cost minimi*" OR "cost evaluation" OR "cost consequen*" OR "cost identificat*" OR "cost analy*" OR "cost containment" OR "cost stud*" OR "cost comparision" OR "quality adjusted life year*" OR "qualityadjusted life year*" OR "disability adjusted life year*" OR "disabilityadjusted life year*" OR "daly*" OR "qaly*" |
| 12 | ALL: "modeling" OR "modelling" OR "model* based" OR "model* technique*" OR "decision analy* model" OR "markov model*" OR "simulation model*" OR "system dynamic model*" OR "discrete event simulation*" OR "microsimulation" OR "agent based model*" OR "cohort model*" OR "decision tree*" OR "probabilit*" OR "transition" OR "patient level simulation" |
| 13 | #10 AND #11 AND #12 |
| 14 | #13 AND NOT Population Group: Animal NOT Editoral NOT PsycTest |

NB: ti: title; ab: abstract; kw: keywords, it: indext term, ALL: any field

| **Name of database: Web of Science (Core Collection)** | |
| --- | --- |
| # | Searching strategies |
| 1 | TS=("mental health" OR "mental well being" OR "mental wellbeing" OR "mental well-being" OR "psychologic* well being" OR "psychologic* wellbeing" OR "psychologic* problem*" OR "psychologic* disorder*" OR "psychologic* disturbance*" OR "mental disease*" OR "mental disorder*" OR "mental illness*" OR "psycho*") |
| 2 | TS=("aggression"OR"aggressive*"OR"anger"OR"mood"OR"emotion*"OR"stress*"OR"obsess*") |
| 3 | TS=("antisocial disorder*" OR "anti social disorder*" OR "antisocial behavio*" OR "anti social behavio*" OR "antisocial personality disorder*" OR "anti social personality disorder*"OR "anti-social disorder*" OR "anti-social behavio*" OR "anti-social personality disorder*") |
| 4 | TS=("anxiety"OR"depress*"OR"conduct disorder*"OR"behavio* disorder*"OR"behavio* problem*") |
| 5 | TS= ("social alienation" OR "impulsive*" OR "impulsivity" OR"isolation") |
| 6 | TS=("conscious*" OR "selfesteeem" OR "self esteem" OR "self-esteem" OR "self efficacy" OR "self-efficacy" OR "selfefficacy") |
| 7 | TS=("suicide" OR "bully" OR "bullying" OR "maltreatment"OR "abuse" OR "neglect*" OR "violence" OR "adverse childhood experienc*"OR"ACE*") |
| 8 | OR #1-#7 |
| 9 | TS=("promot*" OR "prevent*" OR "health education" OR "prevention and control" OR "early intervent*") |
| 10 | #8 AND #9 |
| 11 | TS=("economic evaluation" OR "economic analy*" OR "health economic*" OR "value for money" OR "value-for-money" OR "cost benefit*" OR "cost-benefit*" OR "costbenefit*" OR "cost effective*" OR "cost-effective*" OR "costeffective*" OR "cost efficien*" OR "cost-efficien*" OR "costefficien*" OR "cost utilit*" OR "cost-utilit*" OR "costutilit*" "cost minimi*" OR "cost-minimi*" OR "costminimi*" OR "cost evaluation" OR "cost consequen*" OR "cost identificat*" OR "cost analy*" OR "cost containment" OR "cost stud*" OR "cost comparision" OR "quality adjusted life year*" OR "quality-adjusted life-year*" OR "qualityadjusted life year*" OR "disability adjusted life year*" OR "disability-adjusted life-year*"OR"disabilityadjusted life year*" OR "daly " OR "qaly*") |
| 12 | TS=("modeling" OR "modelling" OR "model* based" OR "model-based" OR "model* technique*" OR "decision analy* model" OR "markov model*" OR "markov-model*" OR "simulation model*" OR "system dynamic model*" OR "discrete event simulation*" OR "discrete-event simulation*" OR "microsimulation" OR "micro-simulation" OR "agent based model*" OR "agent-based model*" OR "cohort model*" OR "decision tree*" OR "probabilit*" OR "transition" OR "patient level simulation" OR "patient-level simulation") |
| 13 | #10 AND #11 AND #12 |
| 14 | None |

NB: TS: Searches for topic terms in the Title, Abstract, Author Keywords and Keywords Plus within a record.

| **Name of database: Econlit** | |
| --- | --- |
| # | Searching strategies |
| 1 | "mental health" OR "mental well being" OR "mental wellbeing" OR "mental well-being" OR "psychologic* well being" OR "psychologic* wellbeing" OR "psychologic* problem*" OR "psychologic* disorder*" OR "psychologic* disturbance*" OR "mental disease*" OR "mental disorder*" OR "mental illness*" OR "psycho*" |
| 2 | "aggression" OR "aggressive*" OR "anger" OR "mood" OR "emotion*" OR "stress*" OR "obsess*" |
| 3 | "antisocial disorder*" OR "anti social disorder*" OR "antisocial behavio*" OR "anti social behavio*" OR "antisocial personality disorder*" OR "anti social personality disorder*" OR "anti-social disorder*" OR "anti-social behavio*" OR "anti-social personality disorder*" |
| 4 | "anxiety" OR "depress*" OR "conduct disorder*" OR "behavio* disorder*" OR "behavio* problem*" |
| 5 | "social alienation" OR "impulsive*" OR "impulsivity" OR "isolation" |
| 6 | "conscious*" OR "self esteem" OR "self esteem" OR "self-esteem" OR "self efficacy" OR "self-efficacy" OR "selfefficacy" |
| 7 | "suicide" OR "bully" OR "bullying" OR "maltreatment" OR "abuse" OR "neglect*" OR "violence" OR "pressure" OR "adverse childhood experienc*" OR "ACE*" |
| 8 | OR #1-#7 |
| 9 | "promot*" OR "prevent*" OR "health education" OR "early intervent*" |
| 10 | #8 AND #9 |
| 11 | "economic evaluation" OR "economic analy*" OR "health economic*" OR "value for money" OR "value-for-money" OR "cost benefit*" OR "cost-benefit*" OR "costbenefit*" OR "cost effective*" OR "cost-effective*" OR "costeffective*" OR "cost efficien*" OR "cost-efficien*" OR "costefficien*" OR "cost utilit*" OR "cost-utilit*" OR "costutilit*" OR "cost minimi*" OR "cost-minimi*" OR "costminimi*" OR "cost evaluation" OR "cost consequen*" OR "cost identificat*" OR "cost analy*" OR "cost containment" OR "cost stud*" OR "cost comparision" OR "quality adjusted life year*" OR "quality-adjusted life-year*" OR "qualityadjusted life year*" OR "disability adjusted life year*" OR "disability-adjusted life-year*" OR "disabilityadjusted life year*" OR "daly*" OR "qaly*" |
| 12 | "modeling" OR "modelling" OR "model* based" OR "model-based" OR "model* technique*" OR "decision analy* model" OR "markov model*" OR "markov-model*" OR "simulation model*" OR "system dynamic model*" OR "discrete event simulation*" OR "discrete-event simulation*" OR "microsimulation" OR "micro-simulation" OR "agent based model*" OR "agent-based model*" OR "cohort model*" OR "decision tree*" OR "probabilit*" OR "transition" OR "patient level simulation" OR "patient-level simulation" |
| 13 | #10 AND #11 AND #12 |
| 14 | None |

| **Name of database: Embase** | |
| --- | --- |
| # | Searching strategies |
| 1 | "mental health'/exp OR "mental health':ti,ab,kw,de OR "mental well being":ti,ab,kw OR "mental wellbeing":ti,ab,kw OR "psychologic* well being":ti,ab,kw OR "psychologic* wellbeing":ti,ab,kw OR "psychologic* problem*":ti,ab,kw OR "psychologic* disorder*":ti,ab,kw OR "psychologic* disturbance*":ti,ab,kw OR "mental disease"/exp OR "mental disease":de OR "mental disease*":ti,ab,kw OR "mental disorder*":ti,ab,kw OR "mental illness*":ti,ab,kw OR "psycho*":ti,ab,kw |
| 2 | "aggression"/exp OR "aggression":ti,ab,kw OR "aggressive*":ti,ab,kw OR "anger"/exp OR "anger":ti,ab,kw OR "mood":ti,ab,kw OR "emotion*":ti,ab,kw OR "stress*":ti,ab,kw OR "obsess*":ti,ab,kw |
| 3 | antisocial personality disorder'/exp OR 'antisocial behavior'/exp OR 'antisocial personality disorder':ti,ab,kw OR 'antisocial behavior':ti,ab,kw OR 'antisocial behaviour':ti,ab,kw OR 'anti-social behavior':ti,ab,kw OR 'anti-social behaviour':ti,ab,kw) |
| 4 | "anxiety"/exp OR "anxiety":ti,ab,kw,de OR "depression"/exp OR "depression":de OR "depress*":ti,ab,kw OR "conduct disorder"/exp OR "conduct disorder":de OR "conduct disorder*":ti,ab,kw OR "behavior disorder"/exp OR "behavior disorder":de OR "behavio* disorder*":ti,ab,kw OR "behavior problem"/exp OR "behavio* problem*":ti,ab,kw |
| 5 | "social alienation"/exp OR "alienation":ti,ab,kw OR "impulsiveness"/exp OR "impulsive*":ti,ab,kw OR "social isolation"/exp OR "isolation":ti,ab,kw |
| 6 | "consiousness"/exp OR "conscious*":ti,ab,kw OR "self esteem"/exp OR "self esteem":de OR "self-esteem":ti,kw,ab OR "selfesteeem":ti,kw,ab OR "self esteem":ti,kw,ab OR "self efficacy"/exp OR "self-efficacy":ti,kw,ab OR "self efficacy":ti,kw,ab OR "selfefficacy":ti,kw,ab |
| 7 | "suicide"/exp OR "suicide":ti,kw,ab,de OR "bullying"/exp OR "bully":ti,kw,ab OR "bullying":ti,kw,ab OR "maltreatment"/exp OR "maltreatment":ti,kw,ab OR "abuse"/exp OR "abuse":ti,kw,ab OR "neglect"/exp OR "neglect*":ti,kw,ab OR "violence"/exp OR "violence":ti,kw,ab OR "pressure"/exp OR "pressure":ti,ab,kw,de OR "adverse childhood experiences"/exp OR "childhood adversity":de OR "adverse childhood experienc*":ti,kw,ab OR "ACE*":ti,kw,ab |
| 8 | OR #1-#7 |
| 9 | "health promotion"/exp OR "promot*":ti,ab,kw OR "prevention"/exp OR "prevention":de "prevent*":ti,kw,ab OR "health education"/exp OR "health education":de OR "health education":ti,kw,ab OR "prevention and control"/exp OR "prevention and control":ti,kw,ab,de OR "early intervent*":ti,kw,ab |
| 10 | #8 AND #9 |
| 11 | "economic evaluation"/exp OR "economic evaluation":de OR "economic evaluation":ti,kw,ab OR "economic analy*":ti,kw,ab OR "health economic*":ti,kw,ab OR "value for money":ti,kw,ab OR "cost benefit analysis"/exp OR "cost benefit*":ti,kw,ab OR "cost effectiveness analysis"/exp OR "cost effective*":ti,kw,ab OR "cost efficien*":ti,kw,ab OR "cost utility analysis"/exp OR "cost utilit*":ti,kw,ab OR "cost minimization analysis"/exp OR "cost minimi*":ti,kw,ab OR "cost evaluation":ti,kw,ab OR "cost consequen*":ti,kw,ab OR "cost identificat*":ti,kw,ab OR "cost analy*":ti,kw,ab OR "cost containment":ti,kw,ab OR "cost stud*":ti,kw,ab OR "cost comparision":ti,kw,ab OR "quality adjusted life year"/exp OR "quality adjusted life year*":ti,kw,ab OR "qualityadjusted life year*":ti,kw,ab OR "disability-adjusted life year"/exp OR "disability adjusted life year*":ti,kw,ab OR "disabilityadjusted life year*":ti,kw,ab OR "daly*":ti,kw,ab OR "qaly*":ti,kw,ab |
| 12 | "economic model"/exp OR "economic model":de OR "economic model*":ti,ab,kw OR "modeling":ti,kw,ab OR "modelling":ti,kw,ab OR "model based"/exp OR "model* based":ti,kw,ab OR "model* technique*":ti,kw,ab OR "decision analy* model":ti,kw,ab OR "markov model"/exp OR "markov chain" OR "markov model*":ti,kw,ab OR "simulation model*":ti,kw,ab OR "system dynamic model*":ti,kw,ab OR "discrete event simulation*":ti,kw,ab OR "microsimulation":ti,kw,ab OR "agent based model*":ti,kw,ab OR "cohort model*":ti,kw,ab OR "decision tree"/exp OR "decision tree":de OR "decision tree*":ti,kw,ab OR "probabilit*":ti,kw,ab OR "transition":ti,kw,ab OR "patient level simulation":ti,kw,ab |
| 13 | #10 AND #11 AND #12 |
| 14 | #13 AND [humans]/lim |

NB: /exp: exlosion search; :ti,ab,kw: search in title, abstract and keywords, /de: search disease emtree preffered term (:de when the term is identified)

**Table S3: Reasons for Fultext Exclusion**

| Author (Years) | Title | Reasons for exclusion |
| --- | --- | --- |
| Nystrand, C., et al. (2017) | Cost-offset analysis of social and emotional learning programs for the prevention of externalizing behavior problems: An economic modeling study | Link to other (#88) |
| Foster, E. M. and D. E. Jones (2007) | The Economic Analysis of Prevention: An Illustration Involving Children's Behavior Problems | Not modelling |
| Behan, C., et al. (2019) | Early intervention in psychosis: health economic evaluation using the net benefit approach in a real-world setting | Not mental health prevention |
| Nystrand, C., et al. (2018) | Indicated Parenting Interventions and Long Term Outcomes: A Health Economic Modeling Study | Link to other (#202) |
| Cadilhac, D. A., et al. (2015) | The health and economic benefits of reducing intimate partner violence: an Australian example | Not full economic evaluation study |
| Dewa, C. S. and J. S. Hoch (2014) | When could a stigma program to address mental illness in the workplace break even? | Not full economic evaluation study |
| McCrone, P., et al. (2013) | The economic impact of early intervention in psychosis services for children and adolescents | Not full economic evaluation study |
| Pil, L., et al. (2012) | Cost-effectiveness analysis of a helpline for suicide prevention | Link to other (#1569) |
| Salomon, J. A., et al. (2012) | Intervention strategies to reduce the burden of non-communicable diseases in Mexico: cost effectiveness analysis | Not mental health prevention |
| Bonin, E. M., et al. (2011) | Costs and longer-term savings of parenting programmes for the prevention of persistent conduct disorder: a modelling study | Not mental health prevention |
| Park, A., et al. (2011) | Multiple economic evaluations of Early Detection (ED) and Early Intervention (EI) for young people with first episode psychosis | Only abstract |
| Valmaggia, L. R., et al. (2009) | Economic impact of early intervention in people at high risk of psychosis | Not full economic evaluation study |
| Ahern, S., et al. (2018) | A cost-effectiveness analysis of school-based suicide prevention programmes | Not modelling |
| Ruby, A., et al. (2013) | Economic analysis of an internet-based depression prevention intervention | Not full economic evaluation study |
| Ulfsdotter, M., et al. (2015) | A Cost-Effectiveness Analysis of the Swedish Universal Parenting Program All Children in Focus | Not Modelling |
| Cadilhac, D. A., et al. (2011) | The societal benefits of reducing six behavioural risk factors: an economic modelling study from Australia | Not full economic evaluation study |
| Cougnard, A., et al. (2005) | A decision analysis model to assess the feasibility of the early detection of psychosis in the general population | Not full economic evaluation study |
| Wellander, L., et al. (2016) | Does Prevention Pay? Costs and Potential Cost-savings of School Interventions Targeting Children with Mental Health Problems | Not mental health prevention |
| Sheehan, P., et al. (2017) | Building the foundations for sustainable development: a case for global investment in the capabilities of adolescents | Included many interventions (mainly mental health treatment, having violence intervention but not related to mental health outcome and not report BCR for this intervention |
| Mohseninejad, L., et al. (2013) | Value of information analysis from a societal perspective: a case study in prevention of major depression | Focused on the methodology of modelling |
| Chisholm, D. and S. Saxena (2012) | Cost effectiveness of strategies to combat neuropsychiatric conditions in sub-Saharan Africa and South-East Asia: mathematical modelling study | Not mental health prevention |
| Aos, S., et al. (2004) | Benefits and costs of prevention and early intervention programs for youth | Did not have interventions directly related to mental health and risk factors |
| Dunlap, L. J., et al. (2019) | Screening and intervention for suicide prevention: a cost-effectiveness analysis of the ED-SAFE interventions | Not modelling |
| Mihalopoulos, C., et al. (2009) | Is early intervention in psychosis cost-effective over the long term? | Not mental health prevention |
| Ssegonja, R. (2020) | Effectiveness and cost-effectiveness of indicated preventive interventions for depression in adolescents: An application of health economics methods | Link to other (#5590) |
| Watmuff, C. and E. Ross (2016) | Costs and benefits of MST-CAN in Leeds: A preliminary internal report to the Chief Officer Children’s Services | Can not find full text, only internal papers linked to #2799 |
| Kass AE et al. (2017) | The economic case for digital interventions for eating disorders among United States college students | Not full economic evaluation study |
| Lokkerbol, J. (2015) | Rationalization of Innovation: The role of health-economic evaluation in improving the efficiency of mental health care | Link to other (#1585) |
| Iijima, S., et al. (2013) | Cost-benefit Analysis of Comprehensive Mental Health Prevention Programs in Japanese Workplaces: A Pilot Study | Not modelling |
| Reynolds, A. J., et al. (2011) | Age 26 cost–benefit analysis of the child‐parent center early education program | Focusing on early education with educational impact, not mental-heath consequences or any risk factor per se |
| Chanley, S. A., et al. (2001) | Providing Refuge: The Value of Domestic Violence Shelter Services | Not prevention strategy but services for domestic violennce victim |
| Dalziel, K. and L. Segal (2012) | Home visiting programmes for the prevention of child maltreatment: cost-effectiveness of 33 programmes | Focused on maltreatment in general, having several outcome not linked to mental health per se |
| Dev, R., et al. (2020) | An economic evaluation of a mobile text messaging intervention to improve mental health care in resource-poor communities in China: a cost-effectiveness study | Focused on patients with schizophrenia |
| Greenwood, P. W. (2004) | Cost-effective violence prevention through targeted family interventions. Youth Violence: Scientific Approaches to Prevention. | A review of methods |
| Vogel, W. B., et al. (2021) | Cost-Effectiveness of the Wellness Incentives and Navigation (WIN) Program | Focused on Texas Medicaid enrollees with co-occurring physical and mental health conditions or severe mental illness (e.g., schizophrenia, bipolar disorder) alone |
| Lokkerbol, J., et al. (2021) | Design of a health-economic Markov model to assess cost-effectiveness and budget impact of the prevention and treatment of depressive disorder | Focused on methods of developing model, not presented prevention intervention in a package of interventions |
| Akers, L., et al. (2017) | Cost-effectiveness of achieving clinical improvement with a dissonance-based eating disorder prevention program | Not a model-based study. Only modeled cost |

**Table S4: Summary of cost-effectiveness results in the included studies**

| **Author (Year) Country** | **EE characteristic** | **Intervention vs. Comparator** | **ICER (in 2020 US$ value)** | **Threshold** | **Sensitivity analysis** | **Author Conclusion** | **Author Specified Limitations** | **Conflict of Interest** | **Source of funding** |
| --- | --- | --- | --- | --- | --- | --- | --- | --- | --- |
| **Depression** |  |  |  |  |  |  |  |  |  |
| Lee (2017) Australia | CUA (Health, Education, 10 years) | Group-based psychological intervention vs. No intervention | AU$ 7,350 ($5,645) /DALY averted (universal); AU$19,550 ($15,015) $AU/DALY averted (indicated) | AU$50,000/DALYs averted | Univariate, PSA | system level implementation of interventions presents value for money | Focused on health benefit linked to the prevention of incident depression; excluded evidence from RCTs measuaring changes in depression symptom in term of continuous scale; assumed that intervention lead to the reduction in depression incidence based on outcomes from RCTs | No | Australian Government National Health and Medical Research Council |
| Mihalopoulos (2011) Australia | CUA (Health, Payer, 5 years) | psychological intervention (bibliotherapy, group base) vs. do-nothing | AU$8600 ($9303) (Brief bibliotherapy) AU$20 000 ($21635) (group therapy) | $AU50,000/DALYs averted | Univariate, PSA | Both interventions show good value for money | Limitations due to assumptions; lack of data for depression epidemiological parameters; not addressed benefits of treating sub-syndromal depression | No | National Health and Medical Research Council |
| Paulden (2010) UK | CUA (Health, 1 year) | Routine screening for postnatal depression + psychologycal therapy vs. usual post-natal care | lowest ICER = £41,103/QALY ($74,419) (Edinburgh postnatal depression scale (EPDS), cutoff point = 16) | £20,000 -30,000/QALY | PSA | Routine screening for postnatal depression does not present value for money to the NHS | Limited published data available for estimating risk of replase, utility weights are not for posnatal women; only EPDS has sufficient data to produce pooled estimate for senstivity and specificity; QALY might not sensitive enough; heterogenety between studies accross all cut points of the considered instruments | No | NIHR Health Technology Assessment Program |
| Hunter (2014) UK | CUA (Health, 1 year) | Screning with a Risk Algorithm (PredictD) + low-intensity prevention program vs. Treatment as usual | £9,607/QALY ($16,603) (PredictD versus TAU) £83,356/QALY ($142,900) (Universal versus TAU) | £20,000/QALY | PSA | PredictD+prevention intervention shows to be cost-effective | Borrowed effectiveness from other systematic review on different interventions; modelled based on low participant rate of UK population; not specified the interventions being modelled; short time duration | No | National Institute for Health Research, Medical Research Concil |
| Lokkerbol (2014) Netherlands | CUA, CBA (Health, 5 years) | Preventive telemedicine vs. a health system with usual care only based on Dutch clinical guidline on depression treatment | ROI=1.45 (without preventive tele); ROI=1.76 (increase preventive tele); ROI=1.77 (increase preventive tele + decrease curative) | €20,000-80,000 /DALY averted | PSA | Offering preventive telemedicine at a large scale is likely to improve cost-benefit ratio | Used value of €20,000/DALY averted rather than the frequently used €50,000/DALY averted; only based on population aged 18-65; not included costs of extending the use of telemedicine but in "steady-state" assumption | No | Netherlands Organization for Health Research and Development; National Institute of Mental Health |
| Mihalopoulos (2012) Australia | CUA (Health, other sector, 5 years) | screening + psychological intervention vs. do-nothing | AU$5400 ($5841) | $50,000/DALYs averted | Univariate, PSA | Screening children for signs of depression and the provision of a psychological intervention to prevent a diagnosable case of MDD represents very good value for money | Borrowed many parameters for adult depression; data on proportion of children scored above 22% CED-S was dated | No | National Health and Medical Research Council (NHMRC) health services research grant |
| van den Berg (2011) Netherlands | CUA (Societal, 5 years) | Opportunistic screening + minimal contact psychotherapy vs. current practice: not screen, not receive minimal contact psychotherapy but recieve care as usual | €1,400 (healthcare) cos-saving (societal) | € 20,000/DALY averted | PSA | the intervention may be cost-effective | Limiations due to assumptions (i.e. 1 year effectiveness of intervention); disability weight of sub-threshold depression based on small sample of clinicians; no adjustment for risk of relapse/recurrence based on disease history; can not assess uncertanty around replapse probability; health care costs of those recovered from depression | No | Ministry of Health, Welfare and Sport in Netherlands |
| Goetzel (2014) US | ROI (Payer, 1 year) | Workplace health risk management program vs. No intervention | ROI = 2.03 | NA | None | could produce meaningful reductions in employee health risks and whether a dollar value could be attached to those reductions | Depended on a pre- and post-test assessment of health risk changes; used national health risk norm as a proxy comparision group; not address the selection bias; used external norms for medical and wage data; assumed unhealthy workers be immediately replaced by healthy ones for each risk category; not consider the level of exposure to the HRM program; not differentiate program components; underestimated program cost; unmeasured factors may have contributed to risk reductions; relied on self-reported data | No | Pinnacol Assurance |
| Leldman (2020) Unclear (but high-income context) | CEA (Societal, 10 years, 5 years) | Group-based cognitive behavivour therapy vs. No intervention | Dominant | Unclear | Univariate, PSA | have a potential to be good value for money | Assumed homogeneous cohort and assumed a constant annual decay rate of the relative treatment effect | Not stated | Not stated |
| Valenstein (2001) US | CUA (Societal, lifetime) | Depression Screening vs. no screening | US$225,467/QALY gained (payer) US$192,444/QALY gained (societal) | $50,000 | Univariate, PSA | Annual and periodic screening for depression cost more than $50 000/QALY, but one-time screening is cost-effective | Wide range of values used for the estimates of several influential model variables; cannot be straightforwardly extended to multidimensional instruments that screen for several psychiatric disorders at once | No | Department of Veterans Affairs Health Services Research and Development Career Development Award |
| Lintvedt (2013) Norway | CUA (Not stated, 1 year) | e-CBT vs. No intervention | NOK$ 3,432/QALY ($505) | NOK$ 500,000 NOK (€67,100) | Univariate | the cost-effectiveness of the translation project was substantial. | Potential bias in validation project; effectiveness based on complete case analyses; effect was considered over 8 weeks but translate to 1 year; outcome estimates were based on self-reports | An author developed of the MoodGYM/BluePages websites | No |
| Jiao (2017) US | CUA (Societal, 50 years) | Depression screening (PHQ-2, PHQ-9) + collaborative care vs. no screening (with usual care) | PHQ 2/9 screening + CC: $1,726/QALY gained ($1,979) | $40,000 | Univariate, PSA | two-stage screening followed by CC was more cost-effective than any other strategy such as screening with PHQ-9 only or screening without CC | Probability of treatment adequacy in CC and the health utility estimates were derived from RCTs in other population; not considered the discountinuation to depression treatment; did not calculate the changes in QALYs for those who do not achieve remission but who did respond partially to the treatments; used triangular distribution; the probability of depression diagnosis and treatment were derived from a general population survey in New York City; excluded depression comorbidity | No | No |
| Premji (2021) Canada | CUA (Health, 2 years) | Screening for depression and follow-up diagnosis and treatment vs. No screening | US$ 17,644 (US$ 18,012) | US$17,321 | Univariate, PSA | The effectiveness and cost-effectiveness of single versus multiple PPD screening opportunities remains an area for further investigation | Unable to explore the cost-effectiveness of intervention for secondary populations (e.g. children) | No | Not stated |
| Ssegonja (2020) Sweden | CEA, CUA (Societal, 5 years, 10 years) | Group based CBT vs. No intervention | Dominant | $20,000 - 100,000 | Univariate, PSA | GB-CBT indicated preventive interventions for depression in adolescents seem to have a potential to be good value for money | Assumed a constant annual decay rate over time; assumed population homogeneity biases in the studies that providing model data inputs; not considered spillover effects and side effects; depression treament not nessessary increase QOL | No | Swedish Research Council |
| **Eating Disorder** |  |  |  |  |  |  |  |  |  |
| Le (2017) Australia | CUA (Health, 10 years) | Cognitive dissonance intervention vs. No intervention | AU$ 103,980/DALY ($70,862) | AU$ 50,000 - 100,000/DALY averted | Univariate, PSA | not cost-effective | Excluded cost outside health sector (e.g. productivity cost that are shown to significant associated with ED); mainly based on the background context of GBD study in Australia -which used a single DW that does not include sub-syndromal disease or disease sererity; the GBD study used a model with many assumption (e.g., a female with AN could never develop BN) to estimate DW | An author sells Ersatz sofware; another receives royalties from publishers | National Health and Medical Research Concil |
| Kass (2017) US | CEA (Payer, 2 years) | Screening+online preventive or treatment vs. wait list | Dominant | NA | None | A stepped care model was estimated to achieve modest cost savings compared to standard care, but these estimates need to be tested with sensitivity analyses | Excluded costs associated with higher levels of care for anorexia nervosa or other eating disorders, medical expenditure result from ED or indirect costs, cost savings of avoiding excess medical utilization through prevention and early intervention; excluded cost of screening | Not stated | National Institute of Mental Health |
| Wang (2011) US | CUA (Societal, 10 years) | School based education and physical activity (Planet Health) vs. usual curricula | Dominant | NA | Univariate, PSA | the Planet Health program is more cost-effective and more cost-saving than previously assessed | Used a single data source for estimation of the long-term medical cost/HRQL; did not include medical costs for the treatment of subdiagnostic BN or travel costs related to treatment of BN; assumed the individuals who were prevented from getting SED would not go on to BN. | No | Leadership Education in Adolescent Health Project, Maternal and Child Health Bureau, HRSA |
| Wright (2014) US | CEA, CUA (Payer, 10 years) | school-based eating disorder screening (and refer to clinician) vs. no screening | US$ 9,041/LY with ED avoided ($ 10,369) US$ 56,500/QALY gained ($ 64,800) | $50,000- 100,000 | Univariate, PSA | Compared with other screening interventions, school-based ED screening may be a cost-effective public health intervention | Assumed that only develop 1 ED subtype during lifetime; some parents maybe unwilling to seek clinical treatment after a positive SCOFF screen; many studies (for input parameters) had small sample sizes and defined EDs inconsistently. | No | No |
| **Anxiety** |  |  |  |  |  |  |  |  |  |
| Ophuis (2018) Netherlands | CUA (Societal, 5 years) | CBT-based early intervetion for subthreshold panic disorder vs. usual care (only intervention for panic disorder patients ) | Dominant | €20,000/QALY | PSA | added CBT-based is cost-saving | Effectiveness based on an RCT with small sample size; did not apply intervention-specific transition probabilities; not considered comorbidity | No | not stated |
| Mihalopoulos (2015) Australia | CUA (Health, 3 years) | Screening and parenting educational program vs. do-nothing | AU$ 8,000/DALY ($6,144) | AU$ 50,000/DALY averted | PSA | intervention bring very good value for money | Efficacy and adherence rate from a single study; no evidence on efficacy in a routine setting; not cover the children with multiple episode in a year; excluded child care cost; borrow parameters from adult population ; only used 3-year duration; not considered parents/siblings' benefit | No | National Health and Medical Research Concil Health Service grant |
| Kumar (2018) US | CUA (Societal, lifetime) | Mobile CBT vs. no CBT or traditional CBT | Dominant (No CBT and traditional CBT) | Unclear | Univariate | Mobile CBT may lead to improved health outcomes at lower costs than traditional CBT or no intervention and may be effective as either prevention or treatment | Not factor the effectiveness of pharmacotherapy in combination with CBT; assumed persons on pharmacotherapy for their entire life; only person in comorbidity states are at increased risk for suicide; mobile CBPT effectiveness from small sample size pilot program; traditional CBT effectiveness from an overall clinical response rate; assumed that both CBT interventions had the same sustained effectiveness; assumed that there are always some residual effects of the CBT programs throughout a person's lifetime | Authors work for Evidation Health; Lantern | Thrive Network, Inc. |
| Simon (2013) Netherlands | CEA (Societal, 2 years) | Screening + early child/parental focused intervention vs. do-nothing | Child or Parent focused was the most cost-effective: €107/AIDS improved child ($13.88) | Unclear | Univariate | Screening+offering child- or parent- focused intervention depending on parental anxiety was the most cost-effective option compared with do-nothing | Based on intermediate outcome measure; main assumptions that lack of information from empirical data; no probabilistic uncertainty analysis; use two-week cost diaires to represent the 1-year cost with small sample size; not considered all alternatives; short time horizon; Markov model is more suitable than decision tree | No | Netherlands organisation for health research and development |
| **Behavior Disorder** |  |  |  |  |  |  |  |  |  |
| Nystrand (2020) Sweden | CBA (Health, Education, until 20 years of age) | group-based indicated parenting programs vs. a wait list control | ROI (trial) =7 (Comet); 10.61 (Connect); 5.96 (Incredible Year); 15.80 (COPE); 328.04 (Self-help book) | NA | Univariate, PSA | all intervention yield substantial social returns and be good value for money | No data from trial for control arm in 2 year follow-up; no sub group analysis; used similar epidemiological trend as trial; unlikely moving below a certain cut-off after intervention would effectively changes the costs; not considered the consequences in adulthood; no cost offsets related to health and wellbeing of caregivers | no | The Swedish Research Concil (2014-10,128) |
| Nystrand (2019) Sweden | CUA (Health, Education, until 18 years of age) | group-based indicated parenting programs vs. a wait list control | US$ 972/DALYs averted ($1,172)  Dominant(Comet) Dominant (Connect) US$224/DALYs averted ($354) (Incredible Year) Dominant (COPE) Dominant (Bibliotherapy) | US$80,000 | Univariate, PSA | All intervention were cost-effectiveness at low willingnes to pay threshold compared with a waitlist control group | Excluded costs from justice and other sectors due to lack of data; not mentioned effect on caregivers' mental health; largely used information from original trial; no transition between CP and ADHP; excluded comorbidity (e.g., oppositional defiant disorder); children' health-outcome are based on parental proxy rather than self-report; assumed the proportion of recovered cases at post-test be the same at follow-up | no | Grants for authors from Swedish Research Council; Swedish National Board of Health and Welfare |
| Mihalopoulos (2007) Australia | CEA (Health, other sector, 26 years) | multi-level system of parenting and family support (Triple P) vs. No intervention | dominant | NA | Univariate | The Triple P Positive Parenting Program is a dominant intervention;likely to be a worthwhile use of limited health funds | Needed to verify assumptions; conduct disorder cost from international literature is not lifetime and exncluded significant cost categories; not included additional benefits (reduction in maltreatment, improvements in parental QoL, and wider societal benefits associated with less social delinquency and crime); Triple P may increase the usage of other services or to reduce service usage | No | No |
| **Other: Psychosis** |  |  |  |  |  |  |  |  |  |
| Wijnen (2020) Netherlands | CUA (Health, 10 years) | CBT-based intervention for Utra-high risk vs. Usual care | Dominant | Unclear | PSA | Conclusion about the usage of PsyMod | Based on limited relevant publication; difficult to apply to other countries; only healthcare perspective; assumed transition probabilities stable over time; 3 stages mentioned in McGorry's modelis simplified as a single post-FEP state; all patients start in the UHR | no | Netherlands Organisation for Health Research and Development Mental Healthcare Fellowship |
| **Risk factor: Suicide** |  |  |  |  |  |  |  |  |  |
| Lebenbaum (2020) Canada | CUA (Societal, 50 years) | Suicide prevention campaigns vs. No intervention | CAD$ 18,853/QALY ($16,916) | CAD$ 50,000 | Univariate, PSA | a suicide-prevention campaign may be cost effective in Ontario | Ignored the high variability of suicide rates and mental healthcare system; used intentional self-harm presenting to emergency departments to estimate suicide attempts; excluded caregivers' costs; not modeled suicide attempts that did not require hospital care; excluded depression state; not incorporated future changes in population structure or economic conditions | A private firm that have pharmaceutical/medical device company as clients | Vanier Canada Graduate Scholarship |
| Kinchin (2020) Australia | ROI (Societal, 5 years) | a school-based gatekeeper training (SafeTALK) vs. Status quo | ROI = 31.2 (Mackay) ROI = 4.1 (Queensland) ROI = 3.3 (Australia) | NA | Univariate | The economic case for implementation of safeTALK is promising on a population basis, especially in high-risk communities | A person could make an attempt/re-attempt immediately after; borrowed meta-analysis from other countries for suicide incidence and re-attempt rates; the coronial inquiry, police, and ambulance costs derived from published literature; assumed that safeTALK does not discriminate based on sex or ethnicity | No | Grapevine Group Mackay and Central Queensland University |
| Denchev (2018) US | CEA (Not stated, 54 week) | Emergency Department-initiated interventions to reduce suicide risk vs. usual care | Dominant (Post card) US$ 4,300/LY saved ($ 4756) (Telephone) US$ 18,800/LY saved ($20,796) (CBT) | $50,000/LY saved | Univariate, PSA | The highly favorable cost-effectiveness found here for each outpatient intervention provides a strong basis for widespread implementation of any or all of these | EDs vary considerably in staffing, treatment protocols, and other characteristics; relied on author opinions to estimate many inputs; not considered improvements in quality of life; largely based on the trials which were conducted under conditions that differ from our model | No | National Institue of Mental Health |
| Richardson (2017) US | ROI (Payer, 30 days) | postdischarge follow-up calls vs. No intervention | ROI=1.76 (comercial insurance) ROI=2,05 (Medicaid) | NA | Univariate, PSA | Proven economical benefit | Uncertainty around the effect size of post-discharge follow-up calls; can not differ the ROI between individuals who express suicidal ideation and who have made suicide attempts; under-coding issues in claim database; also included readmission due to primary mental or substance use disorder rather than only due to suicide | No | Substance Abuse and Mental Health Services Administration |
| Pil (2013) Belgium | CUA (Societal, 10 years) | Suicide helpline vs. no suicide helpline | Dominant | NA | Univariate, PSA | Both telephone- and chat service of suicide helpline are likely to be cost-effective for suicide prevention in Flander | Used the relative risk reduction from an American suicide helpline; it is possible that an individual makes an attempt/re-attempt in the same year; borrowed relative incidence rates for suicide in suicidal persons and for re-attempts from the US and Spain; borrowed annual cost of suicide/patient from the US; based on assumptions in the literature concerning age-related incidences (relative age-dependent incidences of attempts in the Flemish population were also applicable to re-attempts; The age-distribution of the utilities for general health was used to make other utilities age-adjusted) | Not stated | Not stated |
| Comans (2013) Australia | CUA (Societal, 1 year, 5 years) | 24-hr crisis response telephone service vs. usual care | Dominant | $AUD 50,000 | Univariate, PSA | Postvention services are a cost-effective strategy and may even be cost-saving if all costs to society from suicide are taken into account | may be systematic differences between this group of people and all those affected by suicide bereavement; may miss potential benefits accruing as a result of the StandBy program; not able to measure wider costs and benefits to society | Not stated | Not stated |
| Vasiliadis (2015) Canada | CEA (Societal, lifetime) | multimodal suicidal prevention program vs. no program | ICER = CAD$3,979/LY ($3,863) | unclear | Univariate | Suicide prevention program such as NAD trial are cost effective and can result in important potential cost savings | Cost data only based on Quebec data; effectiveness evidence from a study (not RCT); difficult to assess the effect on the reduction of suicide rates attributablee to each modality of suicide prevention programs | No | Quebec Health research fund |
| Godoy (2018) US | CBA (Health, 3 years) | anti-suicide multicomponent program vs. do-nothing | BCR = 4.5 | NA | Univariate | investment may be paid back many times | Excluded related mental health treatment cost; underestimated the program cost; estimated averted health expenditures from secondary sources rather than in the context of the program; suicide attempt reduction rate not from RCT | No | Substance Abuse and Mental Health Services Administration |
| Damerow (2020) Sri Lanka | CEA (Health, other sector, 3 years) | anti-suicide gatekeeper training vs. No intervention | 0.23 fatal cases (maximum of 4.55) needed to be prevented for intervention become cost-effectiveness | $4291.41 | Univariate | The programme needs to prevent an estimated 0.23 fatal pesticide selfpoisoning cases over 3 years to be considered cost-effective | Resource input based on average values derived from expert opinion; possible in-between-shop variations of programme costs; overhead costs of central functions were unknown by the interviewed key informants; excluded spill over effects in the form of cost savings | No | American Foundation for Suicide Prevention; University of Copenhagen |
| Atkins (2013) US | CEA, CUA (Societal, 20 years) | Suicide barrier on the Golden Gate bridge vs. No intervention | ICER = $4,876/DALYs ($5,818) | $48,160 | None | GGB suicide barrier would save lives in a highly cost-effective manner as a result of the reduced lethality of alternate suicide methods in comparison with jumps from the GGB | Not specified limitations (although, the study has no detailed descriptions on model structure; calculated DALY based on asumption that each victims associated with an increase of 37 DALYs; no information on costing methods) | No | Not stated |
| Lee (2020) 14 countires (LIC, HIC) | CUA (Health, lifetime) | Banning highly hazardous pesticides vs. Null comparator | LLMI: $I 94/HLYG UHHIC: $I 237/HLYG | I$ 100/HLYG | Univariate, PSA | potentially cost-effective and affordable intervention for reducing suicide deaths in countries with a high burden of suicides attributable to pesticides | Effect size from observational data; lack of reliable country-specific data; assumed all-cause mortality unchanged; exclude ongoing health-care costs following a suicide attempt; could overestimate intervention costs | No | WHO |
| Martínez-Alés (2021) Spain | CEA (Societal, 1 year) | post-discharge suicide prevention vs. Treatment as usual | Enhanced Contact: €2340 (US$ 3,119) Psychotherapy: €6260 (US$ 8,345) | €28,000-€40,000 | Univariate, PSA | Cost-effective in comparison to a single priority appointment | Effectiveness from an observational study; missed reattempts not receiving hospital treatment and suicide outside of the hospital; not included data on the costs of training providers; untestable assumptions (all potential outcomes distribute similarly across all study centers); only considered the first suicide reattempt | No | Personal training grant |
| **Risk factor: Bullying** |  |  |  |  |  |  |  |  |  |
| Persson (2018) Sweden | CEA, CUA (Payer, 9 years) | school-based anti-bullying program (KiVa) vs. Treatment as usual | SEK 131,321/QALY ($18,812) SEK 7,879/victim-free year ($1,128) | SEK 500,000/QALY | Univariate, PSA | Kiva program may be seen to be cost-effective | Limited data on the effectiveness of the program (no evidence on long term effects; effectiveness of KiVa varies depending on degree of implementation fidelity); there are some activities and costs associated with the status quo activities | one author created and developed Kiva | not stated |
| Beckman (2015) Sweden | CEA (Payer, 3 years) | School-based anti-bullying program (Olweus) vs. no program | SEK 131,250/victim free year ($18,801) | SEK 585,000 | Univariate, PSA | Olweus Bullying Prevention Program is a cost-effective use of resources with a cost per spared victim of bullying equal to 131,250 SEK (V14 470) | Only considered short-term cost and effects; Intervention's efficacy from a relatively small quasi-experimental study | No | No |
| Huitsing (2019) Netherlands | CBA (Societal, lifetime) | School-based anti-bullying program (Kiva) vs. No intervention | ROI=4.04 (Global victimization item) ROI=6.72 (Maximum of 10 specific items) | NA | Univariate | good value for money to invest in KiVa | Assumed all schools maximally implement KiVa; model the cost of alternative is zero since no other validated anti-bullying program; not included other expected benefits on mental health problems; reduced physical problems, improve QoL; only the long-term income benefits for victims were included to the model | No | Dutch Ministry of Education |
| Hummel (2009) UK | CUA (Not stated, lifetime) | anti-bullying program vs. No intervention | £9,600/QALY ($18,345) | £30,000/QALY | Univariate, PSA | Given the uncertainty around effectiveness of an intervention it is recommended that schools monitor victimisation to establish initial levels and whether any interventions they introduce are effective. | The estimation of the effectiveness based on very limited evidence, victimisation prevalence estimation were highly variant | No | No |
| **Risk factor: Violence** |  |  |  |  |  |  |  |  |  |
| Barbosa (2018) UK | CUA (Societal, 10 years) | Identification and referral to improve safety (IRIS) vs. usual care | Dominant | £20,000/QALY | Univariate, PSA | likely to be cost-effective and cost-saving when implemented in the real life | Unable to update the utility value estimates; Many registed women will never experience abuse and therefore cannot directly benefit from the programme; excluded impact of the IRIS programme on children exposed to DVA; used mainly data on short-term outcomes, although modelled long-term outcomes | 2 authors are relating IRIS program | National Institute for Health Research |
| Devine (2012) UK | CUA (Societal, 10 years) | Identification and referral to improve safety (IRIS) vs. no program | Dominant | £20,000-30,000 | PSA | The IRIS programme is likely to be costeffective and possibly cost saving from a societal perspective | Domestic violence prevalence from women attending GP; not include any benefits of the intervention for children who are exposed to DV; trials in this field measure relatively short-term outcomes; the paucity of longitudinal studies measuring the trajectory of abuse and uncertainty about the effect of DV advocacy for women not living in a refuge or shelter | No | Health Foundation |
| Mallender (2013) UK | CUA (Payer , 3 months) | Independence domestic violence advocacy services vs. no program | Dominant | NA | Univariate | prove to be cost-effective | Time horizon only reflected the timeframe within the evaluation study; the study by Howarth is a before-after study which does not provide any control group and based on self report outcome | No | Deliver by Matrix (TMKG limited) as contract with clients |
| Norman (2010) UK | CUA (Societal, 10 years) | a system-based program for better detection and and care for women experiencing intimate partner violence (PreDoVe) vs. control practice (no program) | £742/QALY ($1,417) | £30,000 | Univariate | Cost-effective | Not captured the possibility that women with past IPV histories have a greater likelihood of entering into a new abusive relationship; not differentiated between women in a new abusive relationship and those in longer standing relationships/long term stalking and harassment after leaving an abusive relationship | No | National Institute of Health Research Health Technology Assessment Programme |
| **Risk factor: Abuse** |  |  |  |  |  |  |  |  |  |
| Peterson (2018) US | CBA (Societal, 10 years) | an early education intervention, providing services for low-income family vs. no program | Payer: BCR=0.53 (CPC PO); 0.55 (CPC P+S); 1.79(NFP) Societal: BCR=1.73 (CPC PO); 1.80 (CPC P+S); 6.3 (NFP) | NA | Univariate | Program costs could be substantially offset in the long term through the monetary value of benefits associated with averted CAN | Not up-to-date data on cost; used average program costs per child rather than incremental cost; estimated CPC cost based on small population; local conditions might have affected cost estimates; reference study estimate for the CAN lifetime cost is underestimated; inappropriate assumption of equal effectiveness in a new location; cant not account for the problem that CAN incidence varied by demographic characteristics; excluded states that may already have these programs. | Not stated | Not stated |
| Dopp (2018) US | CBA (Societal, lifetime) | Multisystemic Therapy for Child Abuse and Neglect vs. EOT: a parenting program, received standard outpatient services that were enhanced with study-specific modifications | BRC=3.31 | NA | Univariate, PSA | Demonstrate economic benefits of implementing MST-CAN and create a persuasive argument for increased funding of this treatment model | Could not track all possible outcomes associated with MST-CAN; excluded startup costs (e.g., program development, staff training); unable to take a societal perspective that accounted for costs incurred by families for participation in services (e.g., lost time, travel expenses); the sample size was modest (N = 86) | 2 authors are consultants in the development of MST-CAN | National Institute of Mental Health |
| Kuklinski (2020) US | CBA (Societal, lifetime) | Home visiting interventions vs. Referal calls | BCR: 5.19 to 19.05 | NA | PSA | PFR is cost beneficial assuming tangible victim costs are avoided by PFR. | Not present true population; applied a series of “trumping” rules to avoid possible double counting of benefits from a similar source | No | National Institute of Child Health and Human Development |
